# Design and Synthesis of Small Molecule Probes of MDA-9/Syntenin

**DOI:** 10.3390/biom14101287

**Published:** 2024-10-12

**Authors:** Nehru Viji Sankaranarayanan, Bharath Kumar Villuri, Balaji Nagarajan, Sarah Lewicki, Swadesh K. Das, Paul B. Fisher, Umesh R. Desai

**Affiliations:** 1 Department of Medicinal Chemistry, School of Pharmacy, Virginia Commonwealth University, Richmond, VA 23298, USA; 2 Center for Drug Discovery, Virginia Commonwealth University, Richmond, VA 23219, USA; 3 Department of Human and Molecular Genetics, School of Medicine, Virginia Commonwealth University, Richmond, VA 23298, USA; 4 VCU Institute of Molecular Medicine, School of Medicine, Virginia Commonwealth University, Richmond, VA 23298, USA; 5 VCU Massey Comprehensive Cancer Center, Virginia Commonwealth University, Richmond, VA 23298, USA

**Keywords:** anti-cancer agents, molecular modeling, PDZ domain, protein–protein interactions, small molecule inhibitors

## Abstract

MDA-9/Syntenin, a key scaffolding protein and a molecular hub involved in a diverse range of cell signaling responses, has proved to be a challenging target for the design and discovery of small molecule probes. In this paper, we report on the design and synthesis of small molecule ligands of this key protein. Genetic algorithm-based computational design and the five–eight step synthesis of three molecules led to ligands with affinities in the range of 1–3 µM, a 20–60-fold improvement over literature reports. The design and synthesis strategies, coupled with the structure-dependent gain or loss in affinity, afford the deduction of principles that should guide the design of advanced probes of MDA-9/Syntenin.

## 1. Introduction

Protein–protein interactions (PPIs) play a major role in regulating responses, from cellular growth to death and numerous in between. More than 500,000 PPIs have been hypothesized to occur in humans [1,2], although this could be an over-estimation [3]. A priori, the sheer size of this protein–protein interactome is such that very few common principles of recognition have been enunciated. Generally, PPIs tend to occur over a large surface area (1000–4000 Å^2^) and do not offer well-defined pockets for the engagement of small molecules. Within the large diversity of PPIs, those presented by scaffolding proteins—molecular ‘hubs’ for modulating multiple responses—are most challenging to target. Scaffolding proteins are known to be structurally dynamic to enable multidirectional signal transmission [4,5], which enhances the difficulty of designing small molecule probes of PPIs. In fact, the small molecule disruption of PPIs is considered sufficiently challenging enough to label the constituent proteins as ‘undruggable’.

The melanoma differentiation-associated gene-9 (MDA-9) [6], also called syntenin-1 or the syndecan-binding protein, is one of the key scaffolding proteins that exhibits all the properties of a molecular hub by modulating a diverse range of cellular responses such as migration/metastasis, angiogenesis, lipid/protein trafficking, transcriptional activation, cytoskeletal organization, etc. [7,8,9]. MDA-9/Syntenin was first identified using subtraction hybridization on metastatic human melanoma cells, induced to terminally differentiate and lose cancerous properties [7,8]. Syndecan, the cell surface heparan sulfate proteoglycan identified as the earliest receptor to engage MDA-9/Syntenin [10], forms the basis for its alternate name, the syndecan-binding protein (SDCBP). Aside from syndecan, MDA-9/Syntenin is now known to directly bind to a host of proteins, including c-Src, IGF-1R, TGF-β/TGF-βR, EGFR, PTP-η, neurexin, merlin, IL-5Rα, CD63, and EpHB1/B2.

The primary reason behind MDA-9/Syntenin’s ability to function as a molecular hub is its three-dimensional structure. It is a protein made up of four domains, including *N*- and *C*- terminal domains (NTD and CTD, respectively) and two tandem PDZ domains, which usually contain 80–100 amino acid residues. The latter domains comprise six β-strands and two α-helices that form a distinct globular fold, originally identified in three proteins, namely, post-synaptic density protein 95 (PSD-95), drosophila disc large tumor suppressor protein (DLG), and zona occludens–1 (ZO-1) [11,12].

The *N*-terminal and *C*-terminal domains are fairly disordered and unlikely to be easy to target with small molecules. The crystal structures of the two PDZ domains, labeled PDZ1 and PDZ2, show an overall identical fold, despite their moderate sequence identity (~26%) [12,13,14]. PDZ1 and PDZ2 form the primary docking site for a diverse range of proteins [15,16], which is the reason that MDA-9/Syntenin is classified as a scaffolding protein. In normal situations, this would be called a lack of selectivity; however, for MDA-9/Syntenin, the distinct phenotype that emerges from individual interactions with these proteins results in a highly selective system. Selectivity is also engineered in the interactions of a typical PDZ domain through distinct entropic and structural features that favor certain ligands [17], especially peptides and proteins.

Despite these advantages, targeting MDA-9/Syntenin’s PDZ domains has been challenging. First, the two PDZ domains of MDA-9 are fairly hydrophobic in nature. Second, the two domains together present a rather large PPI surface area. Third, the domains lack well-defined binding pockets, e.g., akin to those of enzymes, that make them increasingly challenging to target [14].

The classic approach to discover lead disruptors of PPIs is to first use peptide sequences of the cognate ligands. In fact, an early attempt utilized hexapeptide epitopes from neurexin and ephrin B. This identified a rather weak, but selective, binding to the PDZ2 domain of MDA-9/Syntenin (100 and 790 µM, see Table 1) [14]. Later, backbone and/or side-chain modifications onto natural peptide epitopes were implemented, as has also been described for several other PPIs [18,19]. For example, Liu et al. took cues from the gain in PSD-95 affinity through dimerization [20] to design a symmetric peptidomimetic dimer derived from a neurexin epitope crosslinked in a head-to-head manner using a tri-ethylene glycol linker. The peptidomimetic displayed the impressive affinity of 201 nM for MDA-9/Syntenin [21]. Recently, a novel peptidomimetic containing cyclohexyl-Gly and *t*-butyl-Gly residues was designed, starting from a heptapeptide identified using phage display technology. The molecule bound MDA-9′s PDZ domains with an affinity for 170 nM, and extended the survival of animals implanted with GBM patient-derived xenografts [22].

Unfortunately, the search for small molecule inhibitors of MDA-9/Syntenin has not been as successful. Using the docking and scoring technique, Leblanc et al. identified an *N*-acetoalkyl-*L*-valine that bound the PDZ2 domain of Syntenin with an affinity of nearly 400 µM (see Table 1). Despite this low affinity, the molecule effectively reduced the exosomal loading of syndecan [23]. Later, the group optimized the structure using crystallography and computational docking to design two analogs that exhibited IC_50_s of 33 and 47 µM [24]. However, further extension of the scaffold led to a significant loss of affinity, implying an uphill task in further drug design. Recently, Tang et al. used ^1^H NMR chemical shift perturbation technology to screen 1430 fragments, and identified four agents with Syntenin PDZ domain affinities in the range of 110 and 610 µM [25].

The most successful small molecule inhibitors of MDA-9/Syntenin to date have been reported by Kegelman et al. [26], Pradhan et al. [27], and Hoffer et al. [28]. Whereas the former reported the discovery of PDZ1i, a linear 1,4-aryldiamine modified with phenyl-oxadiazolyl and triazolo-pyrimidyl groups, through ^15^N/^1^H 2D NMR screening of 5000 fragments and computational modeling, the latter reported on IVMT-Rx-3, a rationally developed polyethylene glycol conjugation from truncated PDZ1i with a PDZ2-binding hexapeptide (TNEYYF). PDZ1i was found to bind primarily in the PDZ1 domain of MDA-9/Syntenin and inhibit EFGR-driven signaling, which reduced FAK signaling and NF-kB activation [27]. PDZ1i also abrogated prostate cancer cell invasion in vitro and metastasis in vivo [29]. Likewise, IVMT-Rx-3 displays an MDA-9/Syntenin affinity of 63 µM (see Table 1), and displays all the characteristics of antimetastatic properties [27]. Finally, a strategy involving molecular dynamics to select key protein conformations that bind syndecan, followed by virtual screening and lead optimization, has led to an MDA-9/Syntenin inhibitor SYNTi with a potency of 400 nM [28].

PDZ1i is a structurally interesting molecule with an excellent biological profile, including EFGR-mediated intracellular signaling and the inhibition of metastasis in vivo [30]. It displays poor aqueous solubility, which necessitates advanced formulation strategies. We reasoned that structural modifications to the PDZ1i scaffold may lead to promising chemical probes that help design lead candidates. Here, we report on the dual-filter genetic algorithm-based computational design and synthesis of three analogs of PDZ1i. Of the three, two analogs exhibited a 20–60-fold increase in affinity, whereas the third designed analog presented a complete loss of MDA-9/Syntenin recognition, alluding to the challenges of targeting this molecular hub. Our design and synthesis strategies, coupled with structure-dependent gain (or loss) in affinity, affords the deduction of principles which could lead to nanomolar small molecule probes/inhibitors of MDA-9/Syntenin.

## 2. Materials and Methods

### 2.1. Computational Design of New Probes

**Figure 1:**
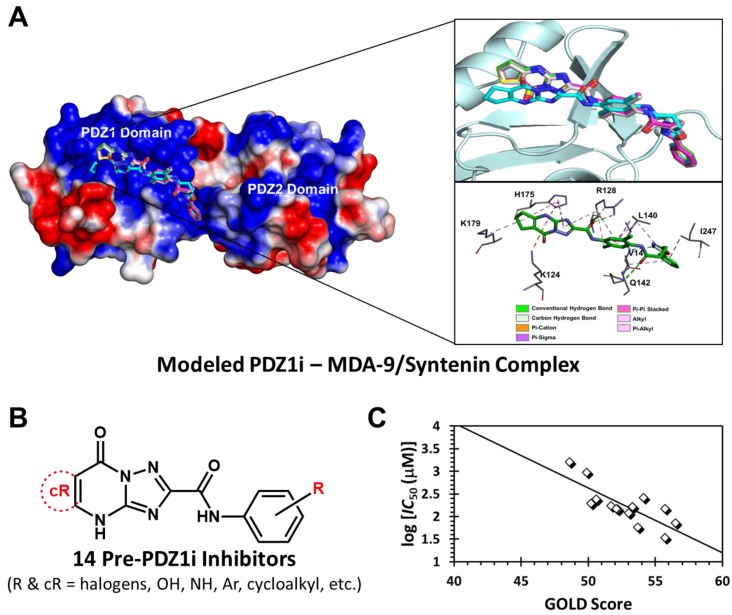
Computational study of 14 synthetic, pre-PDZ1i molecules binding to MDA-9/Syntenin. (**A**) GOLD-docked PDZ1i in the PDZ1 domain of MDA-9/Syntenin. Poses from multiple GA runs in different colors are shown (RMSD = 2.9 Å), which are better revealed in the upper expanded box on the right. The lower expanded box shows the MDA-9 residues predicted to be involved in the binding. (**B**) Structures of PDZ1i and 14 related synthetic analogs. R and cR groups included halogen, hydroxy, amine, aryl, cycloalkyl, and other groups (see Table S1). (**C**) A plot of log IC_50_ v/s GOLD scores for PDZ1i and its analogs. A reasonable correlation coefficient of 0.61 (R^2^) was observed, supporting the applicability of the GOLD-based computational design tool.

The crystal structure coordinates of MDA-9/Syntenin were extracted from the Protein Databank (PDB ID: 1W9E) [14]. The MDA-9 structure was prepared for computational studies using the “Biopolymer” preparation module in SYBYL X21 (Tripos Associates, St. Louis, MO, USA). Hydrogen atoms were first added, and the structure was minimized with fixed heavy-atom co-ordinates using the Tripos force field for a maximum of 5000 iterations subject to a termination gradient of 0.05 kcal/(mol Å). The library of ligands to be computationally investigated was divided into three sets of molecules, including (i) PDZ1i molecule, (ii) 14 synthetic analogs of PDZ1i (see Table S1), and (iii) new designed molecules, which included rational variations in the triazolo-pyrimidyl group (block A), and the 2-aryl-1,3,4-oxdiazole group (block C) (see Table S2). Each ligand molecule was built using the “Molecular sketcher” module in SYBYLX21, and energy minimized (100,000 iterations) using the Tripos force field with Gasteiger–Hückel charges, a fixed dielectric constant of 80, and a non-bonded cutoff radius of 8 Å. These optimized ligand structures were used for further studies. The MDA-9/Syntenin structure has two domains, PDZ1 (residues 114–193) and PDZ2 (residues 198–273), of which PDZ1i was earlier found to engage the former [14]. This binding site was used as the potential binding site for the PDZ1i analogs in our current study. The potential binding sites were mapped using two parameters, namely, cavity depth (SYBLX21) and electrostatic potential (Pymol, APBS tool) (Figure 1A), which led to the docking radius of 16 Å for all experiments. Molecular docking was carried out using GOLD v5.6 [29]. Each structure of the library was docked into the PDZ1i binding site of MDA-9/Syntenin using an unbiased algorithm of 1500 genetic algorithm (GA) runs (with 100,000 iterations). The GOLD Score function, which can be thought of as a surrogate for binding affinity, was used to guide the GA runs. The top six docked poses of each ligand molecule were further analyzed for both binding orientation and consistency of interactions, which were evaluated based on the root mean square deviation (RMSD) between them. GOLD Score and RMSD criteria permit the differentiation of putative ligands for high affinity binding to the PDZ1 domain of MDA-9/Syntenin.

### 2.2. MD of Ligand–Protein Complexes

The best GOLD-docked structures of MDA-9/Syntenin in complex with three ligands (PDZ1i, NGI03, URD001, and NVS125) were prepared for MD using the Leap module of the AMBER18 suite. The ligand molecule was parameterized using the antechamber module, in which AM1-BCC was used to assign the atomic charges. Force field parameters for the three ligands were generated using GAFF. The total charge of the complex was brought to zero by adding an appropriate number of the counter ions. The AMBER-14SB force field was utilized for MDA-9/Syntenin. The charge-neutralized ligand–protein complex was placed in the center of a TIP3P water box with a minimum distance of 12 Å between the box wall and the molecular surface. MD simulations were performed using AMBER18, and periodic boundary conditions were applied to avoid the edge effects [31]. The Particle Mesh Ewald module was employed for calculating long-range interactions. The hydrogen bonds were constrained using the SHAKE algorithm. The ligand–protein complexes were energy minimized in two steps with the non-bonded cut off 10 Å to remove steric hindrance. In the first step, the solute atoms, including the counter ions, were restrained using a harmonic potential with the force constant of 100 kcal/(mol Å^2^). The water molecules were relaxed using 500 cycles of steepest descent and 2000 cycles of conjugate gradient method. In the second step, the whole system was relaxed to conjugate gradient minimization of 2500 cycles without any restrain. 

For MD simulations, the system was equilibrated in three phases including (i) raising temperature to 300 K using the Berendsen temperature coupling with time constant 2 ps, (ii) equilibrating pressure to 1 atm, and (iii) equilibrating all atoms at the NPT without any restrains. All of these phases were performed with in 1 ns. Following this equilibration, a production run of 500 ns was initiated in explicit solvent environment using NPT ensemble with the integration time step of 2 fs. The trajectory files were collected at every 10 ps for further analysis by AmberTools18 [32].

Binding free energy calculation of each protein–ligand complex was performed using the MMGBSA method [33]. The ensemble of structures corresponding to every 20 ps of the dynamic trajectory of the MDA-9/Syntenin–ligand complex was used in the MMGBSA calculations. The MDA-9/Syntenin complexes studied included those with PDZ1i, URD001, NGI03, and NVS125. The energy calculations were performed with the default parameter settings (igb = 5 and saltcon = 0.1), by employing the Python version of the MMGBSA module from AmberTools18. The protocol presented in the amber-MMGBSA tutorial was followed for each calculation (refer to: https://ambermd.org/tutorials/advanced/tutorial3/ (accessed on 20 August 2024)).

### 2.3. Spectrofluorimetry of Ligand–Protein Complexes

Spectrofluorimetric titrations were performed on a Horiba QM400 spectrometer (HORIBA Scientific, Piscataway, NJ, USA). Excitation and emission wavelengths of 280 nm and 340 nm (600 nm for the reference emission wavelength) were used, with 5 nm excitation and 12 nm emission band widths, as described earlier [34]. Experiments were performed in ratiometric mode, with excitation and emission polarizers set to 0,0 angles at room temperature. The titration buffer was 20 mM Tris-HCl buffer, pH 7.4. The stock concentrations of protein and ligands were 40 and 300–1000 μM, respectively. Whereas the protein was prepared in the titration buffer, the ligands were prepared in DMSO. The ligands (either URD001, NGI03, or NVS125) were titrated into the protein solution in aliquots, and saturable changes in fluorescence polarization were used to calculate the affinity. Titrating DMSO into the protein solution (<1% *w*/*v*) did not induce any changes in fluorescence polarization. The emission intensities were corrected for dilution effects (<10% overall). The observed change in fluorescence polarization relative to initial signal (F_0_) was fitted using a standard single-site binding model to obtain the dissociation constant (K_D_) and the maximal change in fluorescence polarization (ΔF) at saturation.

### 2.4. Synthesis of NGI03

#### 2.4.1. Synthesis of Malonohydrazide (**5**)

To a mixture of **4** (50 g (47.2 mL), 1.0 equiv.) in MeOH (500 mL) was added hydrazine hydrate (95.8 g (93 mL, 98% purity), 6.0 equiv.) at 20 °C under N_2_. The mixture was stirred at 80 °C for 12 h, then cooled to 20 °C, and filtered. The cake was washed with MeOH (20.0 mL × 3 times) and concentrated. The filtrate was adjusted to pH 6–8 with 1 M HCl and discarded. Compound **5** was obtained as a white solid (40 g, 97% yield). ^1^H NMR: (400 MHz, DMSO-d_6_) *δ* 9.05 (s, 2H), 4.22 (s, 4H), 2.90 (s, 2H).

#### 2.4.2. Synthesis of N’1,N’3-Dibenzoylmalonohydrazide (**6**)

To a solution of **5** (20 g, 1.0 equiv.) in THF (1.1 L) and K_2_CO_3_ (41.8 g, 2.0 equiv.) in H_2_O (360 mL) at 20–25 °C, was added benzoylchloride (43.8 g (36.2 mL), 2.1 equiv.) dissolved in THF (40 mL) at 20–25 °C and stirred for 12 h. The reaction mixture was slowly added into the ice water (1 L) and the majority of the THF (900 mL) was removed by vacuum. During this process, a number of solids precipitated, which were filtered and dried to obtain **6** as a white solid (39.1 g, 76% yield). ^1^H NMR: (400 MHz, DMSO-d_6_) *δ* 10.36 (s, 4H), 7.95–7.87 (m, 4H), 7.59–7.47 (m, 6H), 3.31 (s, 2H).

#### 2.4.3. Synthesis of Bis(5-phenyl-1,3,4-oxadiazol-2-yl)methane (**1**)

To a solution of **6** (25 g, 1.0 equiv.) in THF (300 mL) was added the Burgess reagent (52.7 g, 3.0 equiv.) at 25 °C warmed to 60 °C and stirred for 12 h. The reaction mixture was then poured into water (1 L), extracted with ethyl acetate (500 mL × 3 times), and the combined organic layers washed with brine (1 L), dried over an. Na_2_SO_4_, filtered, and concentrated under reduced pressure to give the yellow solid **1** (18.3 g, 82% yield), which was used without purification.

#### 2.4.4. Synthesis of tert-Butyl 3,3-bis(5-phenyl-1,3,4-oxadiazol-2-yl)propanoate (**2**)

Compound **1** (5 g, 1 equiv.) was dissolved in THF (50 mL), and 2 M LDA (8 mL, 0.97 equiv.) was added at −20 °C. The mixture was stirred for 3 h and then tert-butyl 2-bromoacetate (3.3 g (2.5 mL), 1.0 equiv.) in THF (15 mL) was added at -20 °C. The mixture was stirred at 25 °C over 12 h under N_2_, quenched with a solution of NH_4_Cl in water (200 mL) and the organic layer separated. The aqueous phase was once again extracted with ethyl acetate (100 mL × 2 times) and the combined organic extracts were dried (an. Na_2_SO_4_), and the solvent was removed in vacuo to give **2** (5 g, crude) as a brown oil. 

#### 2.4.5. Synthesis of N-(4-(3,3-bis(5-phenyl-1,3,4-oxadiazol-2-yl)propanamido)-2,5-dimethylphenyl)-8-oxo-5,6,7,8-tetrahydro-4H-cyclopenta[d][1,2,4]triazolo[1,5-a]pyrimidine-2-carboxamide (NGI03)

To the solution of **2** (5.0 g, 1.0 equiv.) in EtOAc (5 mL) was added 4 M HCl/EtOAc (5 mL), and the mixture stirred at 20 °C for 3 h to obtain 3,3-bis(5-phenyl-1,3,4-oxadiazol-2-yl)propanoic acid. To a solution of this compound (200 mg, 1.0 equiv.) and I-2 (188 mg, 1.0 equiv.) in DMSO (2.0 mL) was added HATU (420 mg, 2.0 equiv.) and DIEA (356 mg (480 μL), 5.0 equiv.) at 0 °C. The reaction mixture was concentrated in vacuo, and purified using C18 prep-HPLC to give NG103 (124 mg, 82% yield, 93.1% purity) as a white solid. LC-MS: Ret. Time = 0.417 min, *m*/*z* (M+1) = 683.4. ^1^H NMR: *δ* 13.80–13.39 (m, 1H), 9.99 (s, 1H), 9.73 (s, 1H), 8.03 (brd, J = 6.8 Hz, 4H), 7.77–7.49 (m, 6H), 7.32 (s, 1H), 7.23 (s, 1H), 5.50 (brt, J = 7.5 Hz, 1H), 3.61 (brd, J = 7.3 Hz, 2H), 2.98 (brt, J = 7.1 Hz, 2H), 2.74 (brt, J = 6.8 Hz, 2H), 2.14 (brd, J = 16.1 Hz, 8H).

### 2.5. Synthesis of URD001

#### 2.5.1. Synthesis of Dimethyl (E)-2-(3-(4-((tert-butoxycarbonyl)amino)-2,5-dimethylphenyl)allyl)malonate (**10**)

A mixture of **7** (4.0 g, 1.0 equiv.) and (Boc)_2_O (4.4 g (4.6 mL), 1.0 equiv.) in toluene (50 mL) was stirred at 100 °C for 10 h under N_2_. LC-MS showed formation of the product (RT = 0.612 min, *m*/*z* = 246.0), which was isolated as a yellow solid using a vacuum, characterized as tert-butyl (4-bromo-2,5-dimethylphenyl)carbamate (5.5 g, 92% yield; LC-MS: RT = 0.612 min, *m*/*z* (M+1) = 246.0), and used without purification.

To the solution of the above compound (4.0 g, 1.0 equiv.), dimethyl 2-allylpropanedioate (8.2 g, 3.6 equiv.), and tris-*o*-tolylphosphane (2.03 g, 0.5 equiv.) in TEA (30 mL), was added Pd(OAc)_2_ (1.52 g, 0.5 equiv.). The reaction mixture was stirred at 90 °C for 12 h. LC-MS showed the formation of a product with an RT of 0.594 min (*m*/*z* = 336.2). The reaction mixture was adjusted to a pH of 4 with 1 M HCl and extracted three times with ethyl acetate (20 mL), organic layer combined and washed with brine (30 mL), and dried over an. Na_2_SO_4_. Following filtering and drying, the residue so obtained was purified using silica column chromatography to give **10** (4.5 g, 86% yield) as a yellow oil (LC-MS: RT = 0.594 min, *m*/*z* (M+1) = 336.2).

#### 2.5.2. Synthesis of tert-Butyl (4-(4-(hydrazinecarbonyl)-5-hydrazineyl-5-oxopentyl)-2,5-dimethylphenyl) carbamate (**11**)

To the solution of **10** (2.0 g, 1.0 equiv.) in MeOH (20 mL) was added Pd/C (500 mg, 10% purity, 1.0 equiv.), and the mixture was stirred under H_2_ (15 psi) at 20 °C for 3 h. The reaction mixture was filtered and the cake washed with MeOH (50 mL), filtered, and concentrated to give dimethyl 2-(3-(4-((tert-butoxycarbonyl)amino)-2,5-dimethylphenyl)propyl) malonate as a yellow oil (1.8 g, 90% yield; LC-MS: RT = 0.622 min, *m*/*z* (M+1) = 338.0).

To the above oil (500 mg, 1.0 equiv.) in EtOH (2 mL) was added hydrazine hydrate (298 mg (290 μL, 85% purity), 4.0 equiv.) at 25 °C. The mixture was stirred at 80 °C for 12 h, then concentrated to give **11** (500 mg, crude), which was a yellow solid. LC-MS: RT = 0.422 min, *m*/*z* (M+1) = 338.0. ^1^H NMR: 400 MHz, CDCl_3_ *δ* 7.51 (br s, 1H), 6.85 (s, 1H), 6.21 (br s, 1H), 2.99–2.91 (m, 2H), 2.54 (br d, *J* = 7.6 Hz, 2H), 2.23 (s, 3H), 2.18 (s, 3H), 1.95–1.87 (m, 2H), 1.51 (s, 9H).

#### 2.5.3. Synthesis of tert-Butyl (4-(4,4-bis(5-phenyl-1,3,4-oxadiazol-2-yl)butyl)-2,5-dimethylphenyl) carbamate (**10**)

To a solution of **11** (500 mg, 1.0 equiv.) and K_2_CO_3_ (352 mg, 2.0 equiv.)dissolved in H_2_O (30 mL) and THF (60 mL) at 20–25 °C, was added benzoyl chloride (375 mg (309 μL) 2.1 equiv.) dissolved in THF (10 mL), and the mixture stirred for 12 h. The mixture was then poured into water (40 mL), extracted three times with ethyl acetate (20 mL), combined, re-washed with brine (50 mL), dried over an. Na_2_SO_4_, filtered, and concentrated to give tert-butyl (4-(5-(2-benzoylhydrazineyl)-4-((2-benzoylhydrazineyl)methyl)-5-oxopentyl)-2,5-dimethylphenyl) carbamate (600 mg, 80% yield) as a white solid. LC-MS: RT = 0.555 min, *m*/*z* (M+1) = 546.4. ^1^H NMR: 400 MHz, DMSO-*d*_6_ *δ* 10.51 (s, 2H), 10.0 (s, 2H), 8.36 (s, 1H), 8.15 (dd, *J* = 1.4, 8.4 Hz, 1H), 7.96–7.91 (m, 1H), 7.91–7.88 (m, 3H), 7.68–7.62 (m, 1H), 7.61–7.54 (m, 3H), 7.53–7.46 (m, 5H), 7.05 (s, 1H), 6.97 (s, 1H), 3.43–3.37 (m, 1H), 2.54 (br d, *J* = 8.0 Hz, 2H), 2.22 (s, 3H), 2.16–2.08 (m, 4H), 1.97–1.85 (m, 2H), 1.68–1.55 (m, 2H), 1.45 (s, 10H).

To a solution of the above compound (2.2 g, 1.0 equiv.) in THF (30 mL) was added methoxycarbonyl-(triethylammonio)sulfonyl-azanide (Burgess reagent) (2.61 g, 3.0 equiv.) at 25 °C, then warmed to 60 °C and stirred for 2 h. The reaction mixture was poured into water (50 mL), extracted with ethyl acetate (30 mL × 3 times), and the combined organic layer washed with brine (150 mL), dried over an. Na_2_SO_4_, filtered, and concentrated under reduced pressure to give a residue, which was purified on silica column gel to yield 3 as a colorless oil (1.0 g, 48% yield; LC-MS: RT = 0.705 min, *m*/*z* (M+1) = 566.2).

#### 2.5.4. Synthesis of N-(4-(4,4-bis(5-phenyl-1,3,4-oxadiazol-2-yl)butyl)-2,5-dimethylphenyl)-8-oxo-5,6,7,8-tetrahydro-4H-cyclopenta[d][1,2,4]triazolo [1,5-a]pyrimidine-2-carboxamide (URD001)

To a solution of **3** (1.0 g, 1.0 equiv.) in EtOAc (5 mL) was added 4 M HCl/EtOAc (5 mL), and stirred at 20 °C for 3 h. The reaction mixture was concentrated under vacuum to afford 4-(4,4-bis(5-phenyl-1,3,4-oxadiazol-2-yl)butyl)-2,5-dimethylaniline as a white solid (700 mg; LC-MS: RT = 0.515 min, *m*/*z* (M+1) = 466.2).

To a solution of the above compound (400 mg, 1.0 equiv.) and **II-1** (284 mg, 1.5 equiv.) in DMSO (6 mL) were added HATU (400 mg, 1.2 equiv.) and DIEA (172 mg (232 μL), 1.55 equiv.) at 0 °C. The mixture was stirred at 25 °C for 2 h, following which H_2_O was added, the mixture extracted with ethyl acetate (200 mL × 3 times), and the organic layers combined, washed with brine (300 mL), dried over an. Na_2_SO_4_, filtered, and concentrated. The residue was purified using C18 prep-HPLC (column: Phenomenex Luna C18 200 × 40 mm × 10 μ) to afford URD001 as a white solid (248 mg, 41% yield, 97% purity; LC-MS: RT = 0.573 min, *m*/*z* (M+1) = 668.4). ^1^H NMR: 400 MHz, DMSO-*d*_6_ *δ* 13.7–13.5 (m, 1H), 9.86 (s, 1H), 7.99 (dd, *J* = 1.6, 8.2 Hz, 4H), 7.71–7.53 (m, 6H), 7.28 (s, 1H), 7.03 (s, 1H), 5.24 (t, *J* = 7.4 Hz, 1H), 3.01–2.95 (m, 2H), 2.76–2.72 (m, 2H), 2.68–2.63 (m, 4H), 2.21 (s, 3H), 2.18–2.08 (m, 5H), 1.77–1.64 (m, 2H).

### 2.6. Synthesis of NVS125

#### 2.6.1. Synthesis of (4-bromo-2,5-dimethylphenyl)methanol (**13**)

To a solution of 1,4-dibromo-2,5-dimethylbenzene **12** (25 g, 1.0 equiv.) in THF (200 mL) was added 2.5 M *n*-BuLi (40 mL, 1.06 equiv.) at −78 °C, and the mixture stirred for 10 min under N_2_. DMF (13.8 g (14.5 mL), 2.0 equiv.) was added to the reaction at -78 °C, before the mixture was allowed to warm up to 25 °C and stirred for 2 h. The mixture was quenched with slow addition of sat. NH_4_Cl solution (400 mL), and the mixture extracted with ethyl acetate (200 mL × 3 times). The combined organic phases were washed with brine (500 mL) and dried (an. Na_2_SO_4_). 4-bromo-2,5-dimethylbenzaldehyde (21 g, crude) was obtained as a yellow solid. ^1^H NMR: 400 MHz, CDCl_3__*d*_6_ *δ*: 7.57–7.76 (m, 1H), 7.43–7.53 (m, 1H), 2.58–2.71 (m, 3H), 2.41–2.49 (m, 3H).

A solution of the above compound (21 g, 1.0 equiv.) in EtOH (200 mL) was cooled to 0 °C, and NaBH_4_ (3.8 g, 1.0 equiv.) was added in small portions over 5 min. The mixture was allowed to reach 25 °C, stirred for another 1 h and poured into water (500 mL). The water layer was extracted with ethyl acetate (300 mL × 3 times), and the combined organic phases were washed with brine (700 mL), dried (an. Na_2_SO_4_), and concentrated in vacuo to give **13** as a white solid (19.2 g, 89% yield). ^1^H NMR 400 MHz, DMSO*___d*_6_ *δ*: 7.33–7.37 (m, 1H), 7.29–7.33 (m, 1H), 5.09–5.15 (m, 1H), 4.37–4.45 (m, 2H), 2.29–2.32 (m, 3H), 2.16–2.20 (m, 3H).

#### 2.6.2. Synthesis of 1-Bromo-4-(bromomethyl)-2,5-dimethylbenzene (**14**)

To a solution of **13** (19.2 g, 1.0 equiv.) in DCM (200 mL) was added PPh_3_ (25.8 g, 1.1 equiv.) at 20 °C, cooled to 0 °C, and then added CBr_4_ (32.6 g, 1.1 equiv.). The mixture was warmed to 20–25 °C, stirred for 12 h, concentrated in vacuo, and purified on silica gel to yield **14** (18.0 g, 72% yield) as a colorless oil. ^1^H NMR: 400 MHz, CDCl_3__*d_6_ δ*: 7.27–7.30 (m, 1H), 7.07–7.11 (m, 1H), 4.32–4.39 (m, 2H), 2.26–2.28 (m, 6H).

#### 2.6.3. Synthesis of 1-(4-Bromo-2,5-dimethylphenyl)-4-(5-(p-tolyl)-1,3,4-oxadiazol-2-yl)butan-2-one (**15**)

To a solution of Zn (13 g, 7.9 equiv.) in DME (15 mL) was added Pd(PPh_3_)_4_ (2.9 g, 0.10 equiv.) at 25–30 °C. Then compound **III-1** (4.4 g, 0.7 equiv.) in DME (15 mL) was added and the solution cooled to 0 °C, following which **14** (7 g, 1.0 equiv.) in DME (15 mL) was slowly added over 0.5 h. The mixture was stirred at 0 °C for 0.5 h, then warmed to 20–25 °C, stirred for 12 h, filtered, concentrated, and purified using silica gel chromatography. The spot (Rf = 0.10) was collected and re-purified using C18 prep-HPLC (column: YMC Triart C18 250 mm × 50 mm × 7 μ; mobile phase: water (FA)-ACN). Compound **15** was obtained as a white solid (560 mg, 54% yield; LC-MS: RT = 2.416 min, *m*/*z* (M+1) = 413.1). ^1^H NMR: EC1995-104-P1A1, 400 MHz, DMSO_*d*_6_ *δ*: 7.84 (*d*, *J* = 8.25 Hz, 2H), 7.36–7.44 (m, 3H), 7.08–7.14 (m, 1H), 3.81–3.91 (m, 2H), 3.08–3.14 (m, 4H), 2.37–2.43 (m, 3H), 2.24–2.29 (m, 3H), 2.07–2.12 (m, 3H).

### 2.7. Synthesis of NVS125

To a solution of **15** (560 mg, 1.0 equiv.) in DMF (20 mL) was added MeOH (5 mL), followed by Pd(dppf)Cl_2_ (200 mg, 0.1 equiv.) and TEA (688 mg (946 μL), 5.0 equiv.) at 25–30 °C. The suspension was degassed under vacuum and purged with N_2_ several times. The mixture was stirred under a CO atmosphere (15 psi) at 25–30 °C, and heated to 90 °C for 24 h. The mixture was then cooled to 25–30 °C and filtered, and the cake so obtained was washed with MeOH (50 mL), with mother liquor collected and concentrated. The residue was purified using silica column chromatography to obtain methyl 2,5-dimethyl-4-(2-oxo-4-(5-(p-tolyl)-1,3,4-oxadiazol-2-yl)butyl) benzoate as a yellow solid (400 mg, 75% yield). LC-MS: RT = 0.552 min, *m*/*z* (M+1) = 413.1. ^1^H NMR: 400 MHz, DMSO_*d*_6_ *δ*: 7.86–7.92 (m, 2H), 7.66–7.70 (m, 1H), 7.44–7.51 (m, 2H), 7.12–7.18 (m, 1H), 3.96–4.01 (m, 2H), 3.83–3.87 (m, 3H), 3.16–3.20 (m, 4H), 2.48–2.51 (m, 3H), 2.44–2.46 (m, 3H), 2.19–2.21 (m, 3H).

To a solution of the above compound (400 mg, 1.0 equiv.) in MeOH (10 mL), LiOH.H_2_O (85 mg, 2.0 equiv.) dissolved in H_2_O (5 mL) was added dropwise at 25 °C warmed to 70 °C and stirred for 4 h. Following the reaction, the mixture was poured into water (30 mL), pH was adjusted to 4–5 with citric acid (20 mL), and the aqueous phase was extracted with ethyl acetate (20 mL × 3 times). The combined organic layer was washed with brine (30 mL), dried (an. Na_2_SO_4_), filtered, and concentrated in vacuo to give a residue, which was purified using prep-HPLC (column: Welch Ultimate C18 150 × 25 mm × 5 μ; mobile phase: [water (FA)-ACN]). NVS125 (73.7 mg, 19% yield, 99% purity) was obtained as a white solid. LC-MS (RT = 0.540 min, *m*/*z* (M+1) = 379.1), ^1^H NMR: 400 MHz, DMSO_*d*_6_ *δ*: 7.80–7.88 (m, H), 7.59 –7.67 (m, 1H), 7.37–7.45 (m, 2H), 7.03–7.10 (m, 1H), 3.93 (s, 2H), 3.07–3.16 (m, 4H), 2.43– 2.47 (m, 3H), 2.38–2.42 (m, 3H), 2.11–2.18 (m, 3H).

## 3. Results

Our rationale for designing advanced ligands started with first assessing whether the genetic algorithm (GA)-based docking and scoring tool GOLD [35] can be used for computationally understanding the interaction of PDZ1i and related molecules with MDA-9/Syntenin. As discussed above, computational simulation of the interaction of small molecules with PPIs is challenging, and we sought to first assess our computational tool. In prior works, we extensively implemented GOLD in designing synthetic mimetics of natural products, especially glycosaminoglycans [36,37], and found it to be particularly useful in segregating selective binders from the promiscuous ones. Hence, we first studied PDZ1i recognition of the PDZ1 site of MDA-9/Syntenin using GOLD-based docking. A moderate level of interaction (GOLD score = 70) was observed for PDZ1i, which was not surprising because its solution affinity was known to be rather modest ~70 µM. However, PDZ1i was found to bind very consistently (i.e., high consistency among bound poses, low RMSD) in the PDZ1 domain of MDA-9/Syntenin, which implied a high level of selectivity (Figure 1A).

Based on the promising results with PDZ1i, we performed the GOLD docking and scoring of 14 molecules (Figure 1B, see also Table S1 in Supplementary Information) that had been studied en route to the discovery of PDZ1i. In silico screening was performed using 100 GA runs in triplicate, and the results presented a range of GOLD scores that correlated reasonably well (R^2^ = 0.6) with MDA-9/Syntenin inhibition potential (log *IC*_50_) (Figure 1C). The best molecule in this group presented a GOLD score of 56.5, while PDZ1i had a score of 70.0, further supporting GOLD as a computational design tool.

**Figure 2:**
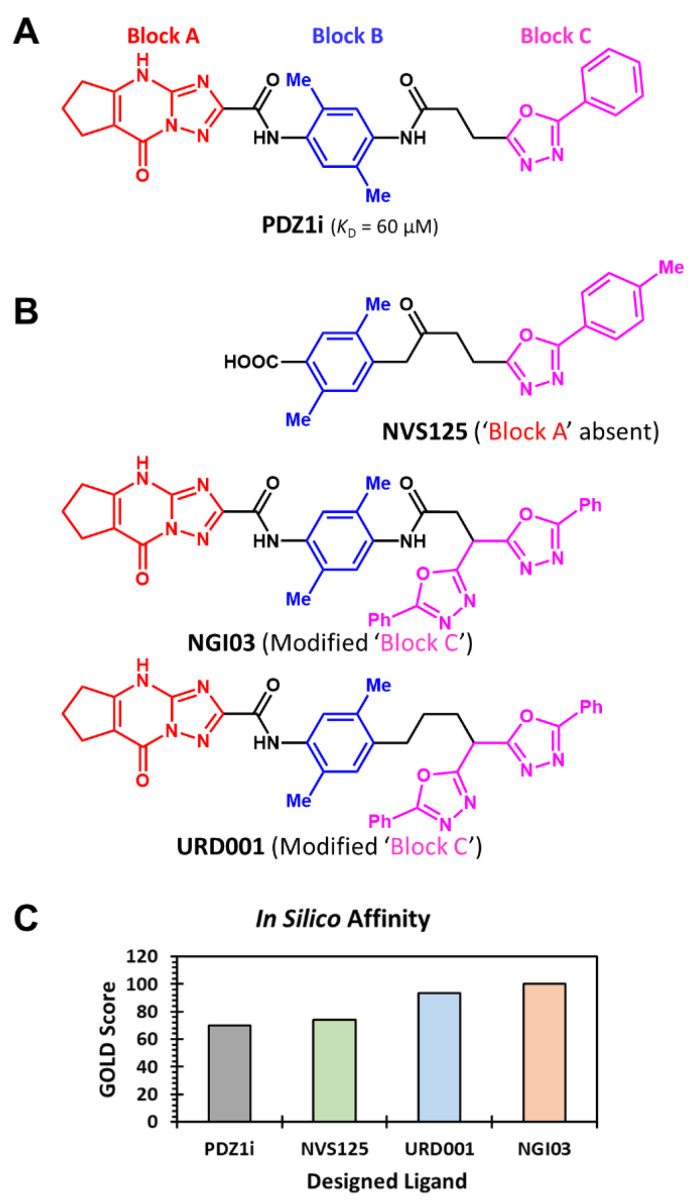
Rationale behind the design of three analogs of PDZ1i. (**A**) The color-coded structure of PDZ1i can be thought of as composed of three major structural blocks, including block A (red), block B (blue), and block C (pink). More specifically, block A corresponds to the triazolo-pyrimidyl scaffold; block B corresponds to the 2,5-dimethyl 1,4-diamino aryl group; and block C to the 2-aryl-1,3,4-oxdiazole group. (**B**) Structures of three PDZ1i analogs NVS125, NGI03, and URD001 were designed as better putative ligands of MDA-9/Syntenin. The primary rationale was to alter blocks A and C, while keeping block B constant. Thus, the design strategy implemented a computational screening of structures containing variations in blocks A and C. Hundreds of variations were studied, from which NGI03, URD001, and NVS125 were selected as computationally promising variants with a predicted higher in silico affinity (GOLD Score). Note: NVS125 does not have block A. In contrast, NGI03 and URD001 have modifications in block C. (**C**) GOLD Score of the four ligands studied in computational screening using GOLD.

In step two, we used GOLD-based docking to design putatively better ligands of MDA-9/Syntenin. We considered PDZ1i (Figure 2A) as being composed of three major structural blocks, including the triazolo-pyrimidyl group (block A), the 2,5-dimethyl 1,4-diamino aryl group (block B), and the 2-aryl-1,3,4-oxdiazole group (block C). We first performed a small docking study of replacing the middle aromatic portion (block B) with non-aromatic cyclic moieties, and found rather low selectivity for the PDZ1 binding site. This implied that modifications should be made in blocks A and C to develop better ligands. Hence, we studied a large library of structures. The GOLD profiles of selected putative ligands are shown in Figure S1 (Table S2 lists structures). A key trend we noted in this study was that ligands designed to be devoid of the triazolo-pyrimidyl group (block A) displayed no improvement in GOLD score. A variation to this principle was noted for NVS125 (Figure 2B), which presented a score of 74.3 (Figure 2C), an improvement of 4.3 over PDZ1i. This increase was small, yet we chose to study NVS125 as the best representative molecule in the group.

Interestingly, when the parent PDZ1i structure was modified to extend the scaffold using aryl or substituted aryl groups, major improvements in GOLD scores were observed. The best structures in this category of designed analogs were URD001 and NGI03 (Figure 2B), which yielded GOLD scores of 93.4 and 100.4, respectively (Figure 2C). These results led us to conclude that NGI03 and URD001 are worth investigating as putative advanced ligands of MDA-9/Syntenin. We also reasoned that NVS125 is structurally a much smaller molecule than PDZ1i/NGI03/URD001, and interesting enough to merit further exploration.

**Figure 3:**
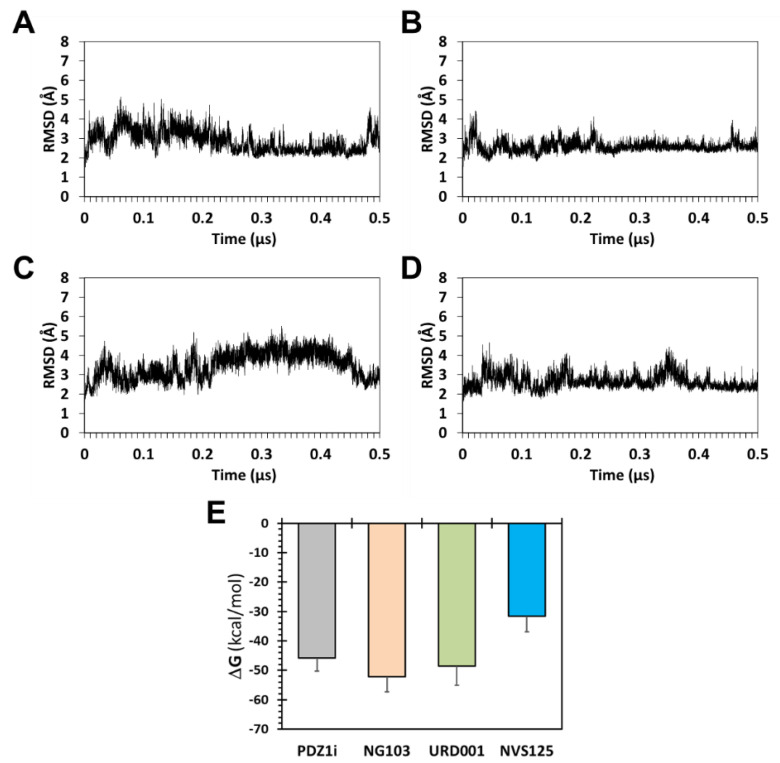
Molecular dynamics simulations of MDA-9–ligand complexes in explicit water (TIP3P box of 12 × 12 × 12 Å^3^). Shown are plots of fluctuations in backbone atoms of MDA-9 in MDA-9–PDZ1i (**A**), MDA-9–NGI03 (**B**), MDA-9–URD001 (**C**), and MDA-9–NVS125 (**D**) over 500 ns of simulation time. Small fluctuations in the backbone of MDA-9 (i.e., low RMSD) suggest stability of the protein–ligand complex in water. Also shown is MD predicted relative in silico binding free energy of four ligands calculated using the MMGBSA tool (**E**). See text for details.

To further test GOLD-based results, molecular dynamics (MD) simulations were performed for MDA-9–ligand co-complexes in a box of water molecules [38]. Comparing the root mean square deviation (RMSD) of backbone atoms of the protein in four MDA-9–ligand complexes over 500 ns of simulation time showed good stability of each ligand in the binding pocket (Figure 3). This conveyed a good probability of retention of interactions in the presence of water and structural dynamism. Interestingly, although URD001 and NGI03 are structurally similar, MD simulations showed an observable difference in their structural mobility in the PDZ1 domain. More specifically, URD001 displayed slightly higher flexibility than NGI03 in the binding pocket (Figure S2).

We also calculated in silico binding energy from the thousands of MD frames using the MMGBSA approach (see Supplementary Information). Although this approach may not yield the solution affinity of ligands, the approach affords analysis of the consistency of atomistic interactions with a target receptor, and is particularly useful to rank order different ligands. Analysis of the ensemble of structures collected every 20 ps over the 500 ns MD run led to the relative ligand affinity order of NGI03 > URD001 > PDZ1i > NVS125 (Figure 3E). These results were in line with the GOLD docking and scoring predictions, although the correlation was not strictly linear.

**Scheme 1:**
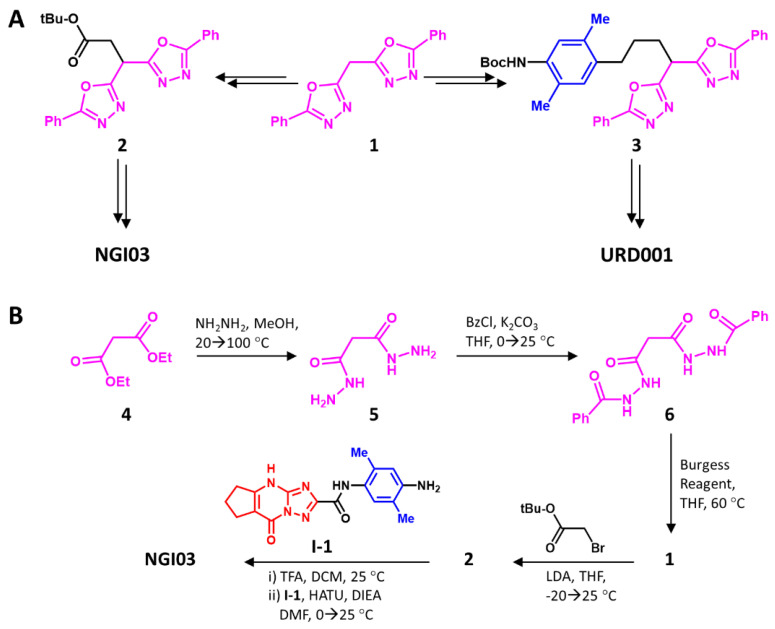
The rationale used in the synthesis of NGI03 and URD001 through a common intermediate (**A**) and the synthesis (**B**) of NGI03. Our rationale was to find a common scheme for both ligands through intermediate **1**. Whereas this approach worked for NGI03, as shown in Panel B, it did not for URD001.

Considering the promise revealed through in silico studies, we embarked on the synthesis of URD001, NGI03, and NVS125. Although the three putative ligands were designed as variants of PDZ1i, a common scheme to synthesize all three molecules was difficult to envisage. Rather, two schemes were projected. The first scheme projected that block C would serve as the common intermediate **1** for both NGI03 and URD001 (Scheme 1). Bis-oxadiazole **1** could theoretically be converted using simple S_N_2 reactions to intermediates **2** and **3**, which eventually would yield NGI03 and URD001, respectively. NVS125 would have to have its own synthetic scheme.

To synthesize intermediate **1**, commercially available diketone **4** was first converted to **6** through bis-hydrazide **5** in good yields (see Methods in Supplementary Information). However, cyclization to **7** using P_2_O_5_, the most common reagent for such reactions [39], failed, despite exploring a range of conditions, especially high temperatures. When the Burgess reagent [40,41] was used, successful conversion to bis-oxadiazole **9** was observed, which led to the ready transformation to **2** under strongly basic conditions. Intermediate **2** was then transformed into NGI03 in two steps, i.e., acidic hydrolysis and basic amidation with pre-synthesized **I-1** (see Scheme S1A), in good yields. The overall yield of the six-step synthesis of NGI03 was an excellent 49% (see Supplementary Information).

**Scheme 2:**
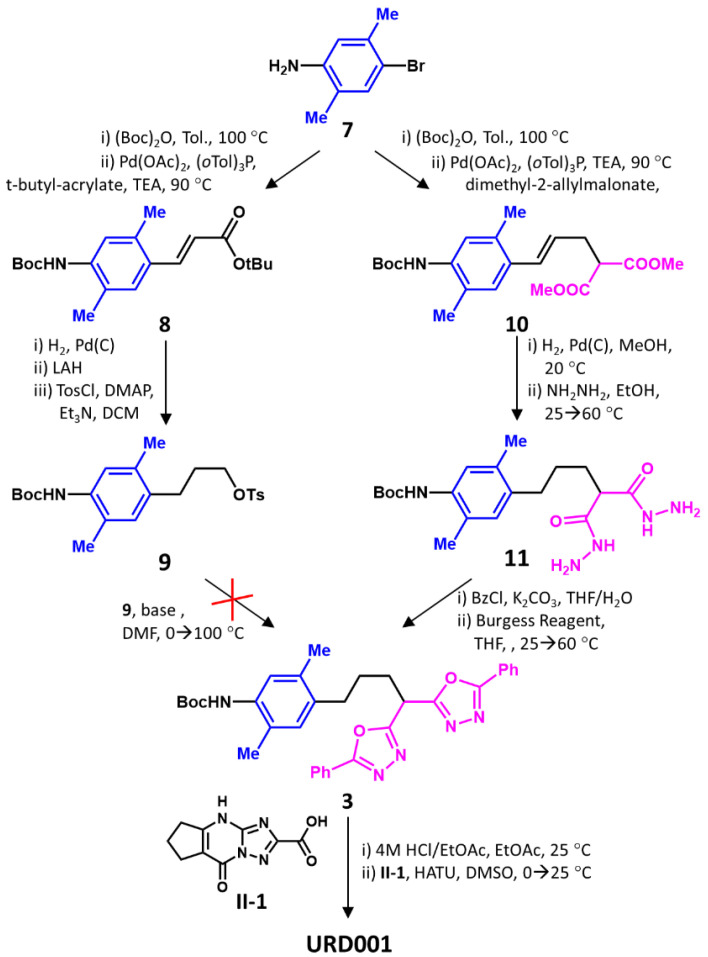
Synthesis of URD001. Although transformation of **7** to **9** in five steps worked out well, nucleophilic displacement of **9**’s tosyl group by **1** under basic conditions did not succeed. Hence, the reverse approach of the bis-oxadiazole group construction from **11** was designed. This involved a four-step conversion of **7** to **11,** followed by a two-step generation of oxadiazole rings, and conjugation with the triazolo-pyrimidyl group **II-1**.

The success of S_N_2-type displacement in NGI03 synthesis led to the expectation that URD001 could also be synthesized in a similar manner. Hence, commercially available **7** was Boc-protected and condensed with *t*-butyl acrylate under Heck conditions [42,43] to yield **8**, which was hydrogenated, reduced, and tosylated to give **9** (Scheme 2). Unfortunately, displacement of the tosyl group in **9** failed. Despite exploring a large number of basic conditions (Cs_2_CO_3_, LDA, etc.), the desired product **3** could not be obtained in more than trace quantities. This was surprising, considering that the *t*-butyl bromoacetate reaction with **1** under S_N_2 conditions was high yielding (Scheme 1). Assuming that the tosyl group was the reason for the failure, we explored the corresponding bromide. Unfortunately, this electrophile also did not yield the desired product **3,** and yielded too many side products.

The failure of the above common scheme led us to explore an alternative strategy. Here, we transformed **7** to unsaturated malonate **10** in two steps, followed by hydrogenation and hydrazinylation to yield intermediate **11** in good yields (Scheme 2). Di-benzoylation and treatment with the Burgess reagent [40,41] yielded **3** in good yields. Acidic de-protection of the Boc group and the HATU-catalyzed coupling of **3** with intermediate **II-1**, which was pre-synthesized as shown in Scheme S1B, gave URD001. Despite the roundabout synthesis, the overall yield of the eight-step synthesis of URD001 was a reasonable 11% (see Supplementary Information).

**Scheme 3:**
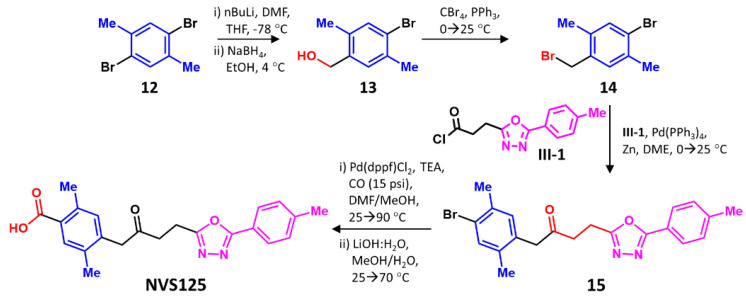
Synthesis of NVS125. This ligand could be synthesized in a relatively straightforward manner in six steps from **12**, wherein intermediate **III-1** had to be pre-synthesized.

Finally, we focused on the synthesis of NVS125 (Scheme 3). Starting with commercially available 1,4-dibromo-*p*-xylene **12**, mono-formylation was achieved with stoichiometric levels of *n*-butyl lithium and DMF, which was followed by borohydride reduction to give **13** in decent yields. The Appel reaction [44] with carbon tetrabromide gave **14**, which was made to undergo the Negishi reaction [45,46] with acyl chloride intermediate **III-1** in the presence of zinc, synthesized in four steps, in the presence of palladium and zinc. **III-1** was pre-synthesized in four steps, as shown in Scheme S1C. Palladium-catalyzed carbonylation of **15** under basic conditions [47], followed by the hydrolysis of the ester so formed, yielded NVS125 in six steps (Scheme 3) with an overall yield of 5% (see Supplementary Information).

To assess the MDA-9/Syntenin binding potential of the synthesized ligands, we studied intrinsic protein fluorescence as a probe of protein–ligand interaction. Unfortunately, no changes in the intrinsic protein fluorescence were observed, probably arising from the lone tryptophan residue in MDA-9/Syntenin. This tryptophan is located 17.5 Å away from the PDZ1 domain, which is perhaps too far to be appreciably affected by the interactions of the ligands.

**Figure 4:**
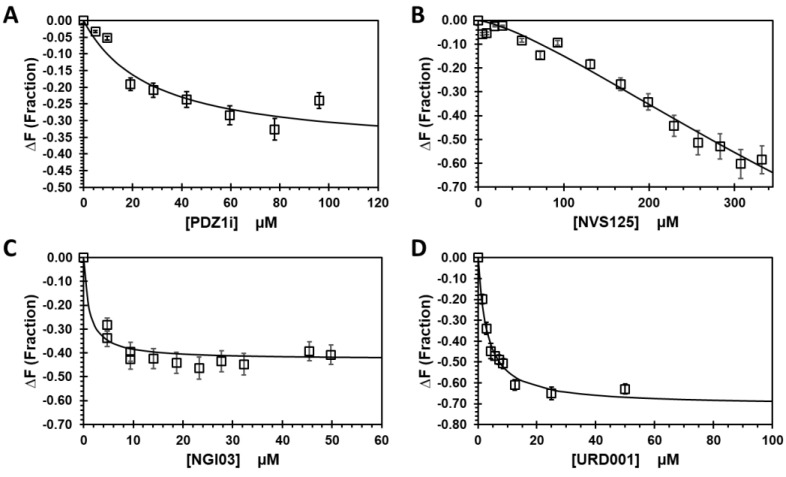
Fluorescence polarization-based measurement of the affinity of four ligands for MDA-9/Syntenin, including (**A**) PDZ1i; (**B**) NVS125; (**C**) NGI03; and (**D**) URD001. A minimum of two titrations were performed and averaged to calculate the K_D_ of interaction. The standard hyperbolic binding isotherm was used to calculate affinity (K_D_) and the maximal change in fluorescence polarization arising from binding (ΔF). See methods for details.

We then explored fluorescence polarization (λ_EX_ = 280 nm, λ_EM_ = 340 nm; λ_REF_ = 600 nm) in 20 mM Tris-HCl containing 100 mM NaCl, 0.01% PEG800, and 0.05% Tween20, pH 7.4. Polarization relies on the change in molecular diffusion of the protein in the presence of a ligand as a probe of interaction. This technique worked well for PDZ1i, which presented a classic hyperbolic binding profile. Non-linear regression using the standard binding isotherm yielded a *K*_D_ of 78 μM (Figure 4). For NGI03 and URD001, *K*_D_s of 1.1 and 3.0 μM, respectively, were measured. In contrast, NVS125 did not yield a well-defined, saturable binding profile (Figure 4B). This implies that no reliable fit of the data could be obtained. Yet, based on the profile, the *K*_D_ is likely to be more than 200 μM. This implies that NVS125 does not bind well to MDA-9/Syntenin.

## 4. Discussion

This work reports the computational design of three analogs of PDZ1i, namely URD001, NGI03, and NVS125. The three analogs were synthesized in six to eight steps, each in reasonable yields and high purity. We measured the affinities of the three agents for MDA-9/Syntenin and compared them with PDZ1i under identical conditions. URD001 (3.0 μM) and NGI03 (1.1 μM) were found to have a much higher affinity than that of PDZ1i (~78 μM) for MDA-9/Syntenin (Figure 4). Alternatively, the rational GOLD-based design effort led to a nearly 24–70-fold improvement in MDA-9/Syntenin affinity. Considering the challenges in discovering small molecule disruptors of PPIs, this bodes well for future design campaigns.

Although successful in terms of URD001 and NGI03, computational design appears to have failed with NVS125. This is not too surprising, because molecular modeling is known to put forward false positives, which may be enhanced due to challenges associated with the particular protein at hand, i.e., MDA-9/Syntenin. Yet, this result underscores the importance of a more rigorous computational approach in the future. Specifically, it would be useful to add a comprehensive MD study that screens multiple initial conformations of lead ligands, rather than only one from the molecular docking study, as done here. Perhaps such a comprehensive study may not only reduce false positives but also not miss false negatives, an aspect that was not evaluated here.

Although URD001 and NGI03 are very promising in terms of affinity, both ligands were found to have aqueous solubility issues. Both molecules required 100% DMSO for biological studies, which is a deterrent in pre-clinical development. Interestingly, NVS125 exhibited good aqueous solubility, which pinpoints the planar triazolo-pyrimidyl group (block A) as a key source of solubility issues. Alternatively, a new design campaign should minutely consider the role of block A in MDA-9 recognition and biophysical properties. In fact, the current results, which highlight some important principles, are likely to be very useful.

## 5. Conclusions

The structure–activity relationship developed in this work for MDA-9/Syntenin–ligand interactions leads to several important conclusions.

One, a key conclusion can be drawn with regard to the small molecule structure–activity relationship (SAR) for PDZ1-containing proteins, especially MDA-9/Syntenin. Introduction of a bifurcated block C enhances MDA-9/Syntenin affinity dramatically. More specifically, the two phenyl-oxadiazolyl groups of NGI03 are observed to bind in the bifurcated channel sandwiched between MDA-9′s PDZ1 and PDZ2 domains (see Figure S3). This observation could help design better chemical probes for MDA-9/Syntenin, as well as for other PDZ domain-containing proteins.

Two, another important SAR conclusion is that the lack of the triazolo-pyrimidyl group, i.e., block A (Figure 2), appears to completely eliminate MDA-9/Syntenin recognition. As described above, the hydrophobic and planar triazolo-pyrimidyl group drastically reduces aqueous solubility. Thus, the triazolo-pyrimidyl group is a double-edged sword. While it appears to engineer selective and high affinity recognition of the PDZ1 domain, it also introduces aqueous solubility challenges. Hence, future efforts may well gain ground by exploring a reduction in the planarity of this ring, which ensures selectivity as well as potency.

Three, several independent schemes had to be developed for the reliable synthesis of three putative ligands of the PDZ domain. Given the role of the bifurcated block C, the synthetic steps developed in this direction are robust, good yielding, and amenable to scale up (Scheme 1 and Scheme 2). Thus, employing this chemistry for molecules containing the bifurcated block C will be particularly easy in the synthesis of advanced PDZ ligands.

Overall, URD001 and NGI03 are structurally promising analogs of PDZ1i. Both ligands are amenable for modification. The current results posit that avoiding solubility (and other physicochemical properties) issues without losing selectivity and potency is an achievable challenge. In fact, this work conveys that genetic algorithm-based virtual screening is likely to be particularly suited to such an effort.

## Figures and Tables

**Table 1:** MDA-9/Syntenin Affinities of Different Ligands Known to Date.

Ligand	MDA-9 *K*_D_ (μM)	Binding Domain	Ref.
*Peptides*			
Neurexin hexapeptide	100	PDZ2	[14]
Ephrin hexapeptide	790	PDZ2	[14]
Neurexin-derived peptidomimetic dimer	0.201	PDZ1, PDZ2	[20,21]
Heptapeptide	0.170	PDZ2	[22]
*Small Molecules*			
*N*-acetoalkyl-*L*-valine	400	PDZ2	[23]
Aryl analogs of *L*-valine	33 and 47	PDZ2	[24]
Four aromatic fragments	110–610	PDZ1, PDZ2	[25]
PDZ1i	60–70	PDZ1	[26]
IVMT-Rx-3	63	PDZ1, PDZ2	[27]
SYNTi	0.400	PDZ1	[28]

## Data Availability

All research data will be shared with any researcher interested in this work.

## Supplementary Materials

The following supporting information can be downloaded at: https://www.mdpi.com/article/10.3390/biom14101287/s1, Figure S1: GOLD scores of 54 designed PDZ1i analogs; Figure S2: RMSD plots of the movement of four ligands in the MDA-9 bound form; Figure S3: GOLD-docked ligands in the PDZ1 domain of MDA-9/Syntenin; Scheme S1: Overall schemes for synthesis of Intermediates I-1, II-1 and III-1; Table S1: Structure, binding affinity and docking properties of pre-PDZ1i inhibitors of MDA-9/Syntenin; Table S2: Structures and GOLD scores of analogs designed based on the PDZ1i structure.

## Abbreviations

CTD, C-terminal domain; DLG, drosophila disc large tumor suppressor protein; GA, genetic algorithm; GBM, glioblastoma multiforme; MDA-9, melanoma differentiation associated gene–9; MST, microscale thermophoresis; NTD, N-terminal domain; PDZ, PSD-95, DLGA, and ZO-1; PPI, protein–protein interactions; PSD-95, post-synaptic density protein 95; SDCBP, syndecan-binding protein; ZO-1, zona occludens–1.
