# Supplementary material for: Design and Synthesis of Small Molecule Probes of MDA-9/Syntenin
Source: Biomolecules. 2024 Oct 12;14(10):1287. doi: 10.3390/biom14101287 (PMC11505911; doi:10.3390/biom14101287)
Supplement: Supplementary file 1 [file biomolecules-14-01287-s001.zip › biomolecules-3207890-supplementary.pdf]

Table of Contents

No.	Title	Pg.
1	Table S1. Structure, binding affinity (or inhibition) and docking properties of pre-PDZ1i inhibitors of MDA-9/Syntenin.	S2
2	Table S2. Structures and GOLD scores of analogs designed based on the PDZ1i structure.	S3
3	Figure S1. GOLD scores of 54 designed PDZ1i analogs and their comparison with the corresponding score for PDZ1i.	S7
4	Figure S2. RMSD plots of the movement of four ligands in the MDA-9 bound form.	S8
5	Figure S3. GOLD-docked ligands in the PDZ1 domain of MDA-9/Syntenin.	S9
6	Scheme S1. Overall schemes for synthesis of intermediates I-1, II-1, and III-1.	S10
7	Detailed procedures for synthesis of intermediate I-1.	S11
8	Detailed procedures for synthesis of intermediate II-1.	S12
9	Detailed procedures for synthesis of intermediate III-1.	S13
10	^{1}H NMR spectra and HPLC Chromatograms	S15

Table S1. Structure, inhibition and docking properties of synthetic pre-PDZ1i inhibitors of MDA-9/Syntenin.

ID	Structure	IC_{50} (μM)	GOLD Score	RMSD (Å)
30A9	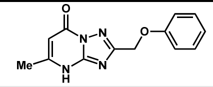	197	50.2	3.9
CMPD11	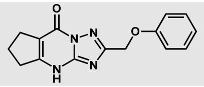	171	51.7	0.5
112G4	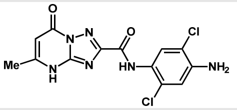	34.3	55.7	2.4
CMPD10	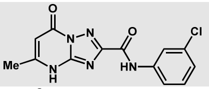	151	55.7	0.4
112D11	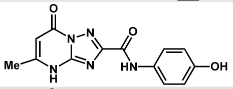	245	50.6	2.5
112E7	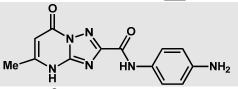	944	49.9	0.4
112G2	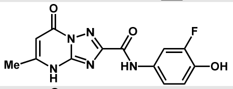	147	52.2	9.9
112G1	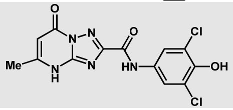	57.3	53.7	10.9
112G11	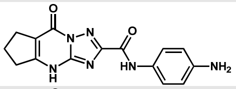	263	54.1	0.3
112E12	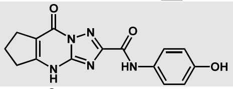	1610	48.6	0.4
113B8	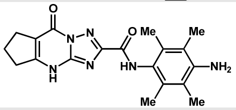	151	52.1	10.1
112F12	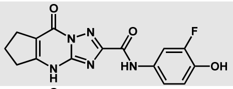	166	53.3	0.6
112G3	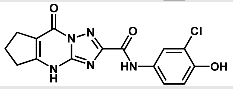	119	53.0	1.3
112F11	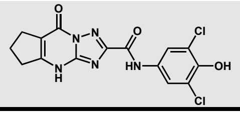	71.8	56.5	0.3

Table S2. Structures and GOLD scores of analogs designed based on the PDZ1i structure.

#	ID	Structure of Designed Analog	GOLD Score	RMSD (Å)
1	NVS1	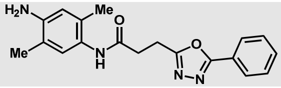	66.2	1.8
2	NVS2	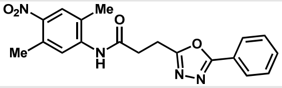	65.0	2.4
3	NVS4	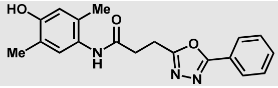	65.1	1.9
4	NVS11	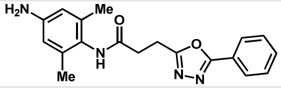	67.7	2.2
5	NVS12	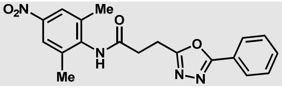	69.4	1.8
6	NVS16	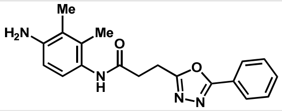	66.5	1.5
7	NVS20	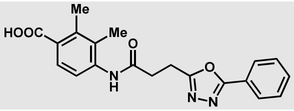	59.9	1.8
8	NVS23	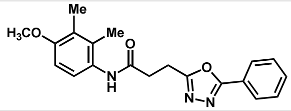	65.7	1.8
9	NVS26	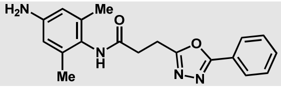	65.8	1.5
10	NVS47	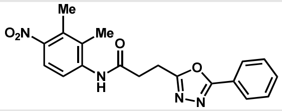	58.2	1.7
11	NVS62	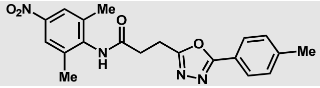	68.9	1.4
12	NVS64	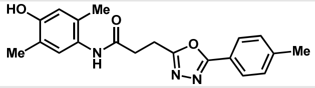	67.1	2.4
13	NVS67	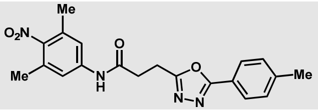	55.7	1.6
14	NVS73	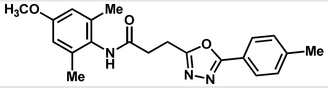	67.9	0.5
15	NVS74	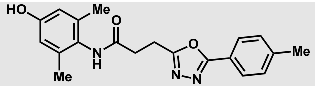	69.1	0.9
16	NVS76	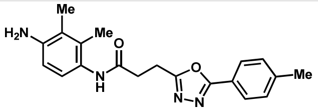	69.3	2.0

17	NVS77	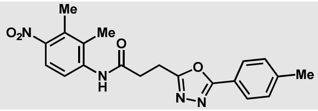	71.1	1.9
18	NVS79	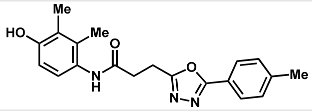	65.6	2.3
19	NVS85	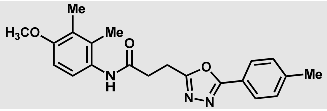	61.3	2.0
20	NVS86	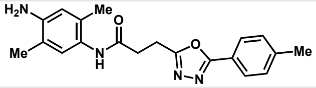	67.1	1.9
21	NVS87	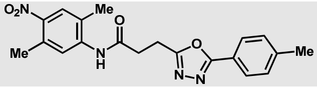	68.0	1.6
22	NVS89	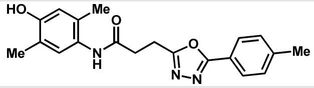	65.8	2.1
23	NVS90	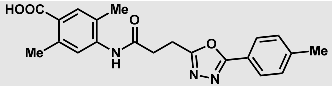	69.0	1.7
24	NVS93	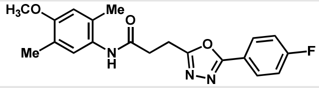	63.8	1.6
25	NVS95	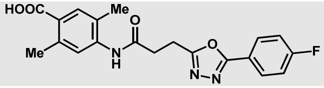	66.9	1.6
26	NVS104	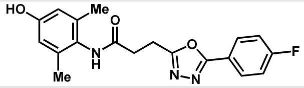	65.3	2.3
27	NVS106	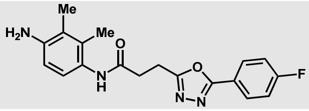	64.8	2.4
28	NVS109	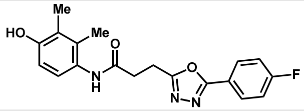	59.9	1.5
29	NVS110	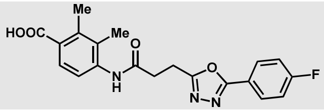	59.3	1.6
30	NVS114	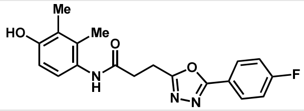	64.0	1.9
31	NVS117	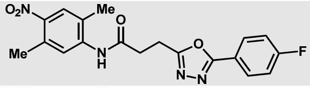	64.9	2.2
32	NVS125	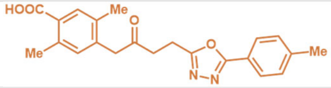	74.0	2.2
33	NVS129	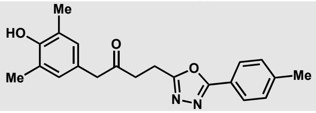	70.2	2.1

34	NVS130	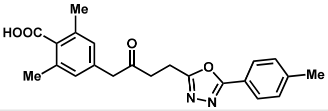	71.9	1.5
35	NVS131	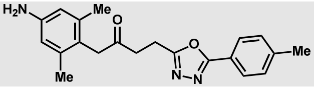	68.5	1.0
36	NVS132	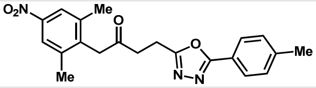	68.5	1.9
37	NVS136	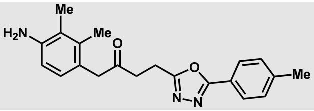	68.1	2.4
38	NVS147	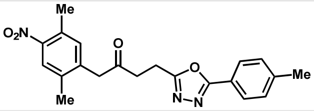	66.2	1.9
39	NVS148	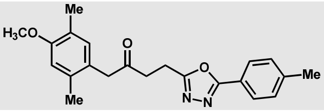	68.1	2.0
40	NVS152	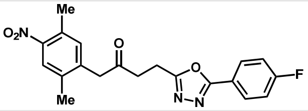	65.8	2.5
41	NVS153	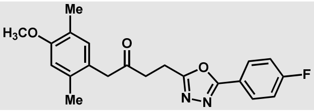	69.3	2.4
42	NVS158	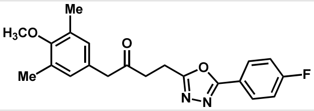	71.0	1.7
43	NVS161	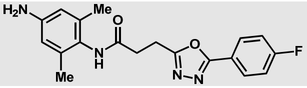	65.2	1.8
44	NVS178	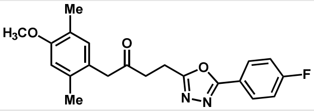	64.7	1.6
45	NVS500	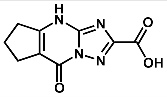	37.0	4.8
46	PDZ1i	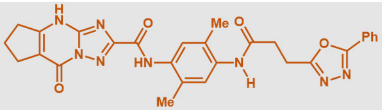	70.0	2.9
47	PDZ1i_a	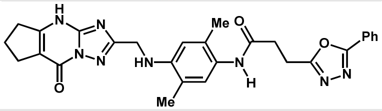	73.8	1.6
48	PDZ1i_b	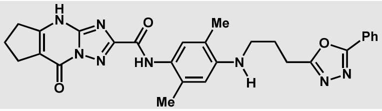	82.3	2.9
49	PDZ1i_c	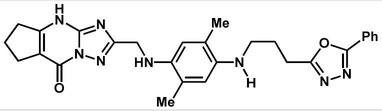	80.0	4.1

50	112H9	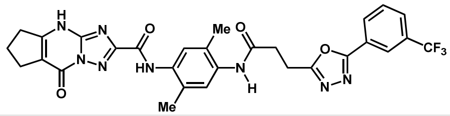	71.5	1.7
51	113B11	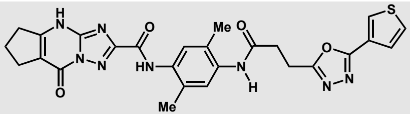	74.0	0.9
52	113B12	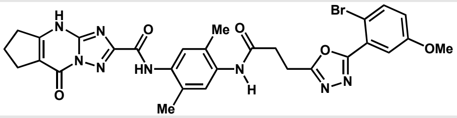	76.8	2.3
53	113B9	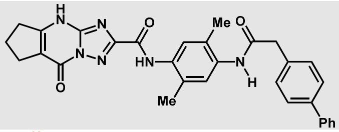	78.3	2.4
54	URD001	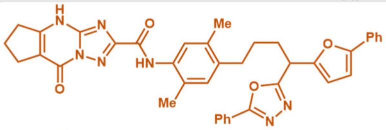	93.4	3.4
55	NGI03	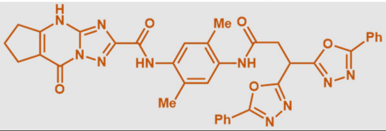	100.4	2.1

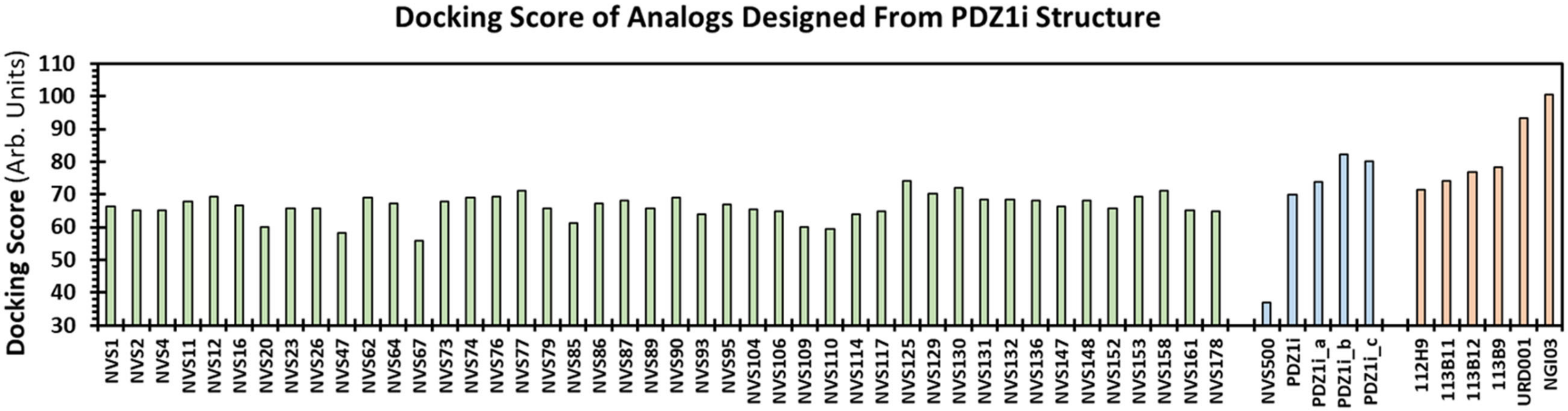

Figure S1. GOLD scores of 54 designed PDZ1i analogs and their comparison with the corresponding score for PDZ1i. Exact structures and docking results are listed in Table S2. Whereas the majority of designed molecules (green bars) mimicking the right half of the PDZ1i structure resulted in moderate GOLD scores (55 to 69), derivatives of the linkers (blue bars) and scaffold extension (orange bars) resulted in high GOLD scores (70 to 100).

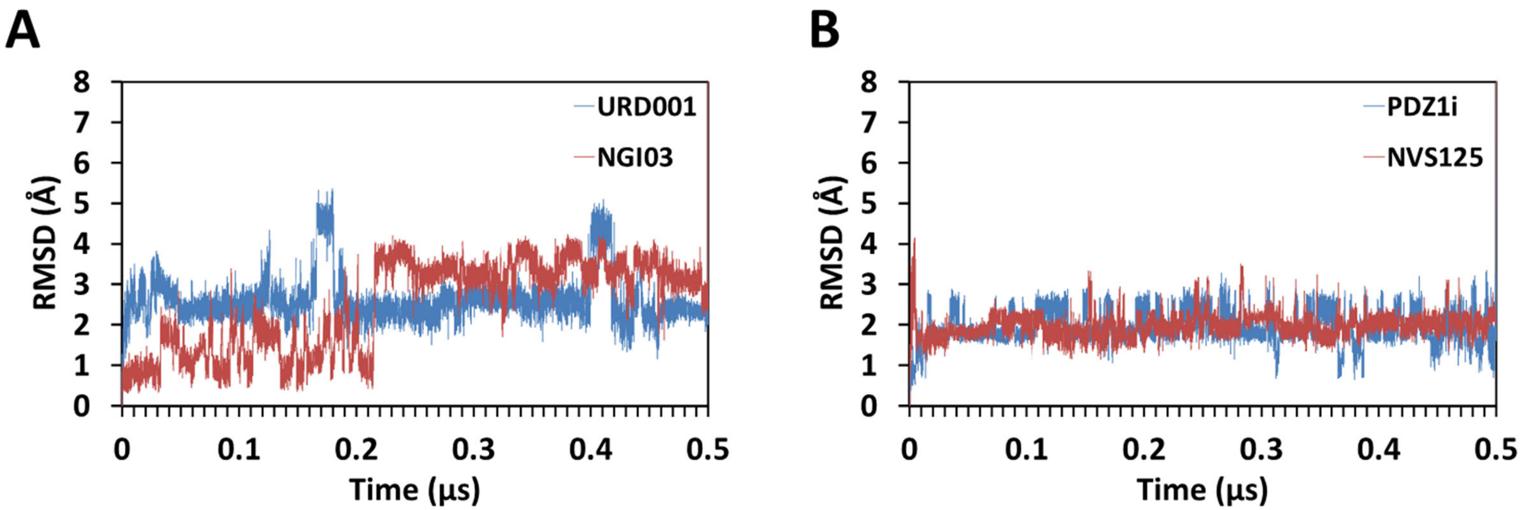

Figure S2. Molecular dynamics simulations of MDA-9—ligand complexes and analysis of fluctuations in non-hydrogen atoms of ligands URD001 and NGI03 (A) and ligands PDZ1i and NVS125 (Panel B). Whereas NGI03, PDZ1i and NVS125 were observed to be relatively consistent and stable in their binding poses throughout MD simulation, URD001 was noted to flip between different conformational states.

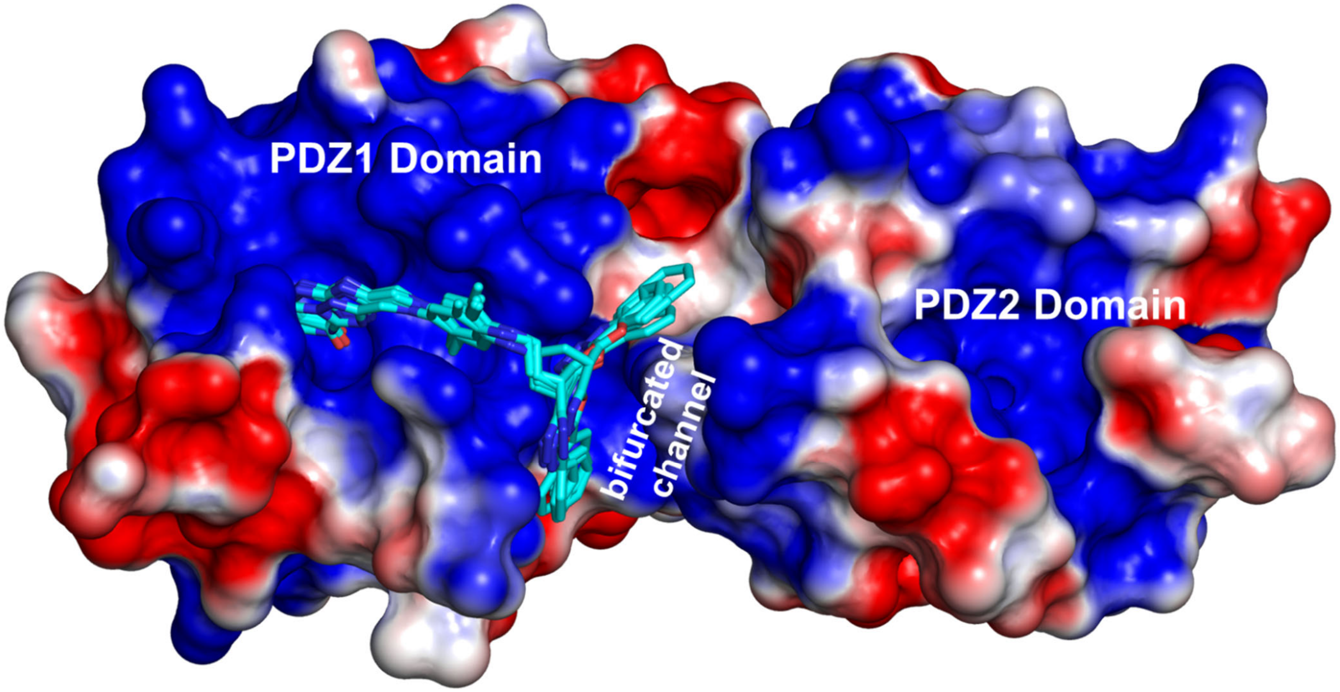

Figure S3. GOLD-docked NGI03 in the PDZ1 domain of MDA-9/Syntenin. Poses from multiple GA runs in different colors are shown. Note the high consistency of binding observed in silico (RMSD<2.5 Å).

(A) Intermediate I-1

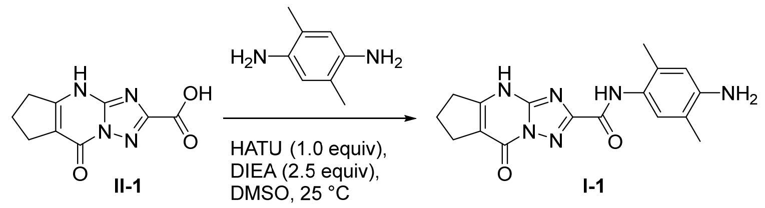

(B) Intermediate II-1

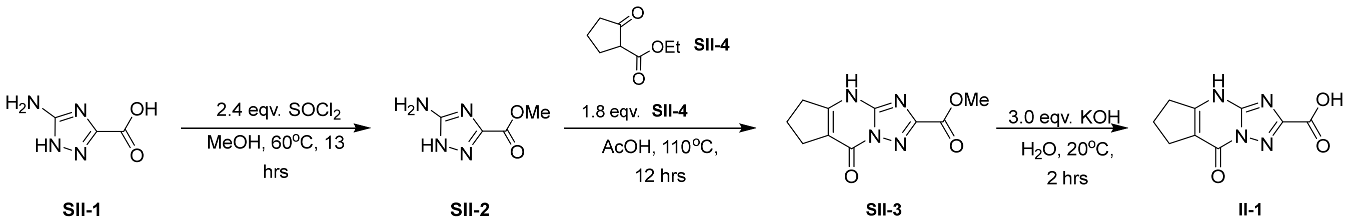

(C) Intermediate III-1

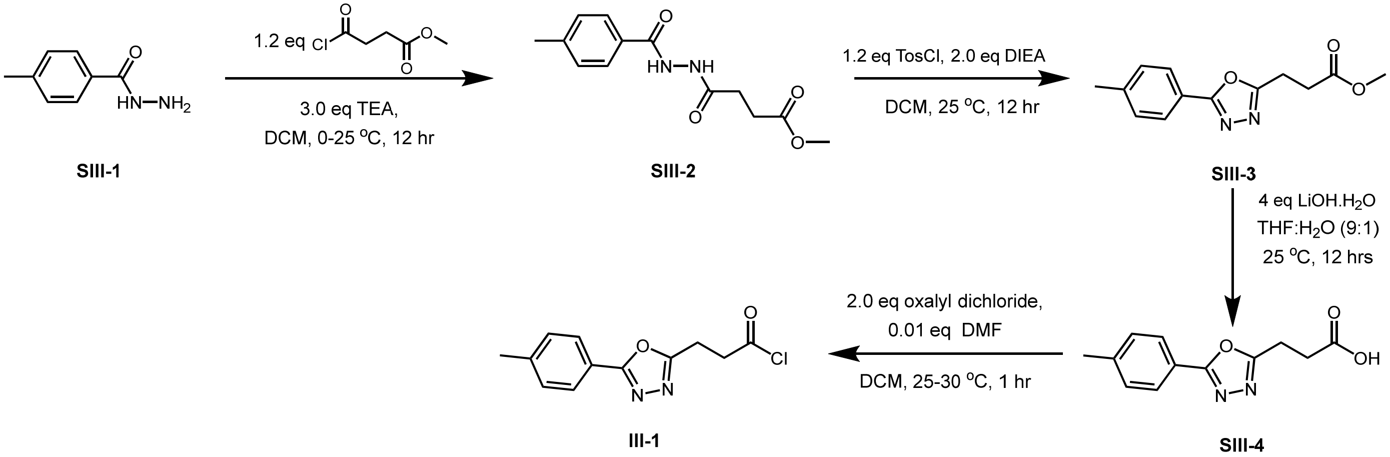

Scheme S1. Overall schemes for the synthesis of key intermediates I-1 (A), II-1 (B), and III-1 (C) used in the synthesis of NGI03 (shown in Scheme 1), URD001 (shown in Scheme 2), and NVS125 (shown in Scheme 3).

Detailed Procedures for Synthesis of Intermediate I-1

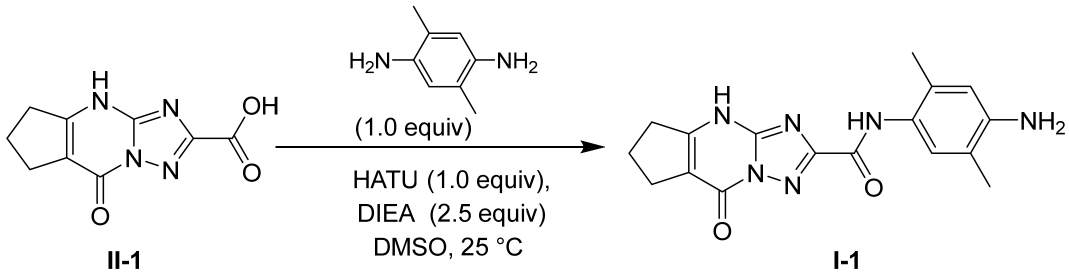

Synthesis of *N*-(4-amino-2,5-dimethylphenyl)-8-oxo-5,6,7,8-tetrahydro-4*H*-cyclopenta [d][1,2,4]triazolo[1,5-a]pyrimidine-2-carboxamide I-1: To a solution of **II-1** (200 mg, 1.0 equiv.) and 2,5-dimethyl-1,4-amino benzene (188 mg, 1.0 equiv.) in DMSO (2.0 mL) was added HATU (420 mg, 2.0 equiv.) and DIEA (356 mg (480 μL), 5.0 equiv.) at 0 °C. The mixture was stirred at 25 °C for 2 h, concentrated in vacuo to give the product.

Detailed Procedures for Synthesis of Intermediate II-1

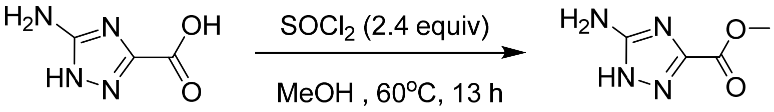

SI-1 **SI-2**

Synthesis of methyl 5-amino-1H-1,2,4-triazole-3-carboxylate (SI-2)—To the solution of **SI-1** (1.0 g, 1.0 equiv.) in MeOH (15 mL) was added $SOCl_2$ (1.0 g (623 μL), 1.2 equiv.) and the mixture stirred at 60°C for 1 h, followed by addition of another batch of $SOCl_2$ (1.0 g (623 μL), 1.2 equiv.) and stirring for 12 h. The reaction mixture was concentrated to obtained **SI-2** (1.2 g, crude) as a yellow solid.

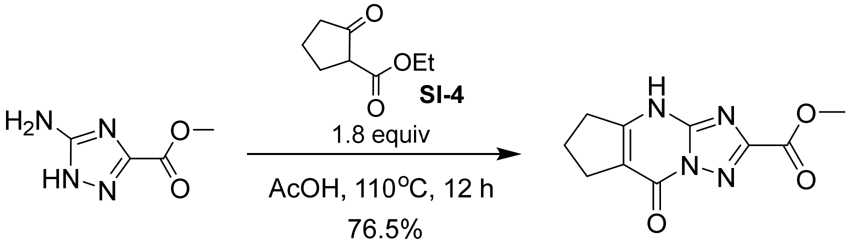

SI-2 **SI-3**

Synthesis of methyl 8-oxo-5,6,7,8-tetrahydro-4*H*-cyclopenta[d][1,2,4]triazolo[1,5-a]pyrimidine-2-carboxylate (SI-3)—The solution of **SI-2** (1.2 g, 1.0 equiv.) and **SI-4** (2.4 g (2.3 mL), 1.8 equiv.) in AcOH (20 mL) was stirred at 110 °C for 12 h. The solution was filtered and washed with petroleum (30 mL) to give **SI-3** (1.4 g, 76% yield) as a yellow solid. **LC-MS:** RT = 0.359 min, m/z (M+1) = 235.2

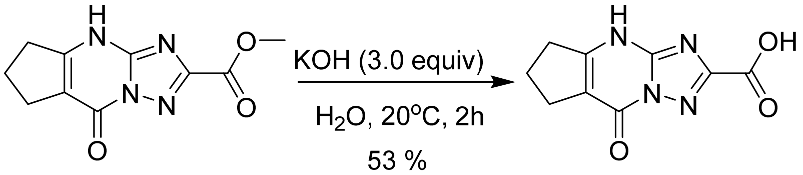

SI-3 **II-1**

Synthesis of 8-oxo-5,6,7,8-tetrahydro-4H-cyclopenta[d][1,2,4]triazolo[1,5-a]pyrimidine-2-carboxylic acid (II-1)—The solution of 2M KOH (4.5 mL, 3.0 equiv.) in H_2O (4.5 mL) was added to **SI-3** (700 mg, 1.0 equiv.). The mixture was stirred at 20 °C for 2 h, then pH adjusted to 4.0 with 1M HCl, followed by filtering and washing with petroleum ether (50 mL) to afford **I-1** (350 mg, 53% yield) as a brown solid. **LC-MS**: RT= 0.237min, m/z (M+1) = 221.1

Detailed Procedures for Synthesis of Intermediate III-1

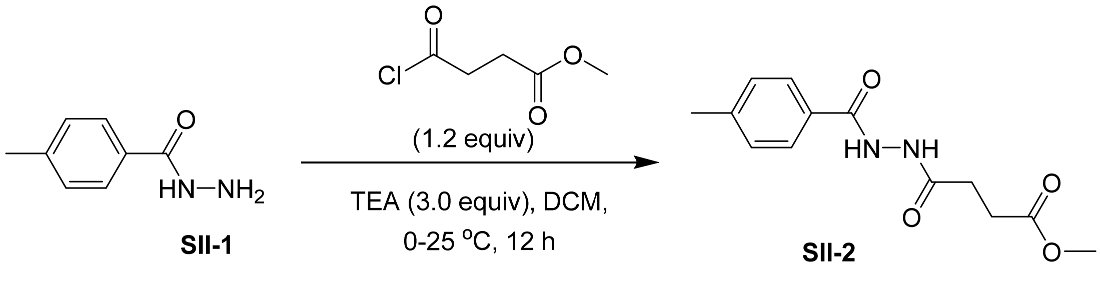

Synthesis of methyl 4-(2-(4-methylbenzoyl)hydrazineyl)-4-oxobutanoate (SII-2)—**SII-1** (20 g, 1.0 equiv.) was solubilized in DCM (200 mL) at 25 °C, then cooled to 0 °C, followed by addition of TEA (40.7 g (56 mL), 3.0 equiv.) and **SII-2** (24 g (20 mL), 1.2 equiv.). The mixture was warmed to 25 °C, stirred for 12 h under N_2 and poured into H_2O (300 mL) followed by extraction with DCM (200 mL × 3 times). The combined organic layer was washed with saturated brine (300 mL), dried (an. Na_2SO_4), filtered, and concentrated in vacuo to give **SII-2** (32.5 g, crude), which was taken to the next step without purification. **LC-MS**: RT = 0.351 min, m/z (M+1) = 265.1 **^{1}H NMR:** 400 MHz, DMSO_d_6 δ: 10.24 (s, 1H), 9.88-9.95 (m, 1H), 7.74-7.79 (m, 2H), 7.27-7.33 (m, 2H), 3.60 (s, 3H), 2.55-2.59 (m, 2H), 2.28-2.42 (m, 5H).

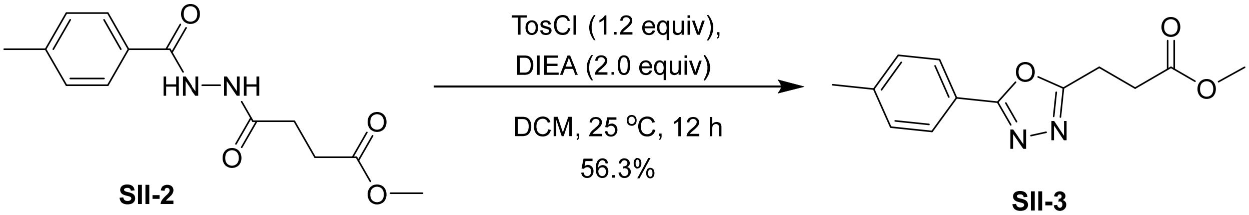

Synthesis of methyl 3-(5-(p-tolyl)-1,3,4-oxadiazol-2-yl)propanoate (SII-3)—To a solution of **SII-2** (32.5 g, 1.0 equiv.) in DCM (350 mL) was added TosCl (28.1 g, 1.2 equiv.) and DIEA (31.9 g (43 mL), 2.0 equiv.*)* at 25 °C. The reaction was stirred at 25°C for 12 h and worked up as usual (DCM extraction, brine washing, an. Na_2SO_4 drying and silica gel purification. **SII-3** (19 g, 56% yield, 90% purity) was obtained as a yellow solid. **LC-MS:** RT = 0.440 min, m/z (M+1) = 247.1

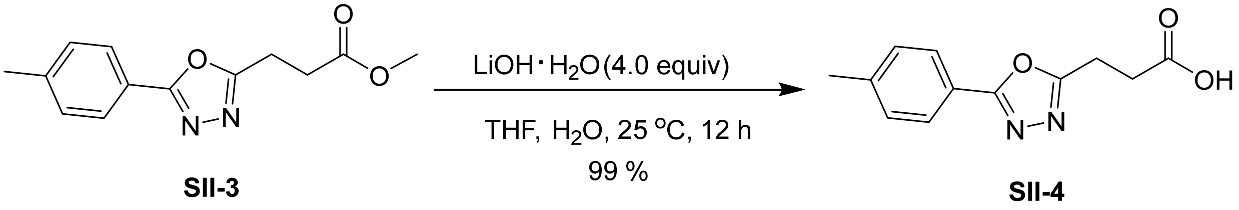

Synthesis of 3-(5-(p-tolyl)-1,3,4-oxadiazol-2-yl)-propanoic acid (**SII-4**)—To a solution of **SII-3** (19 g, 1.0 equiv.) in THF (200 mL) was added LiOH.H_2O (11.6 g, 4.0 equiv.) dissolved in H_2O (20 mL) dropwise at 25 °C and stirred for 12 h. The reaction mixture was worked up as usual to yield **SII-4** (16 g, 99% yield) as a yellow solid. **^{1}H NMR:** 400 MHz, DMSO_d_6 δ: 7.82-7.91 (m, 2H), 7.36-7.46 (m, 2H), 3.09-3.17 (m, 2H), 2.77-2.84 (m, 2H), 2.37-2.41(m, 3H).

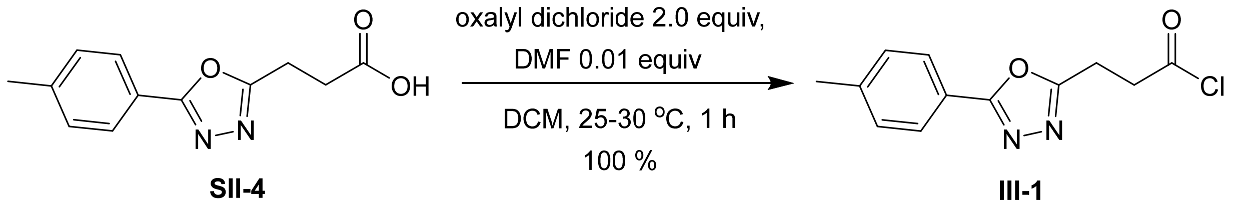

SII-4 → III-1: oxalyl dichloride 2.0 equiv, DMF 0.01 equiv; DCM, 25-30 °C, 1 h; 100 %

Synthesis of 3-(5-(p-tolyl)-1,3,4-oxadiazol-2-yl)propanoyl chloride (III-1)—To a solution of **SII-4** (4 g, 1.0 equiv.) in DCM (40 mL) was added DMF (380 mg (400 μL), 3.0 equiv.) and oxalyl dichloride (4.35 g (3.0 mL), 2.0 equiv.) at 25-30 °C. The mixture was stirred at 25-30 °C for 1 hr and concentrated under vacuum to yield **III-1** (4.32 g, 100% yield) as a yellow solid.

H^1-NMR Spectra for NGI03, URD001 & NVS125

Compound ID: NG103

EC3153-42-P1C1 DMSO Bruker_CD-C_400MHz

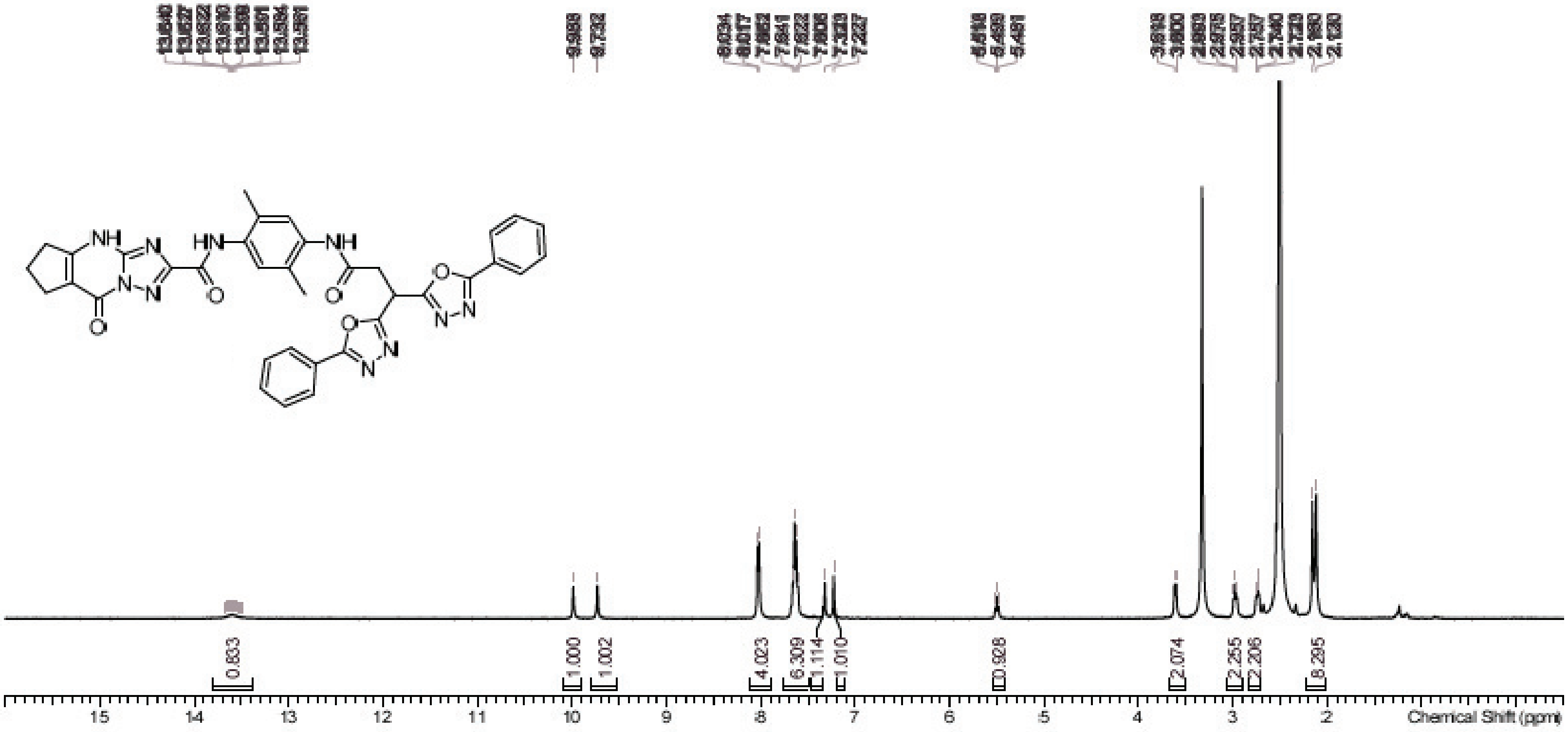

Compound ID: URD001

EC6016-36-P1A1 DMSO Bruker_CD-A_400MHz

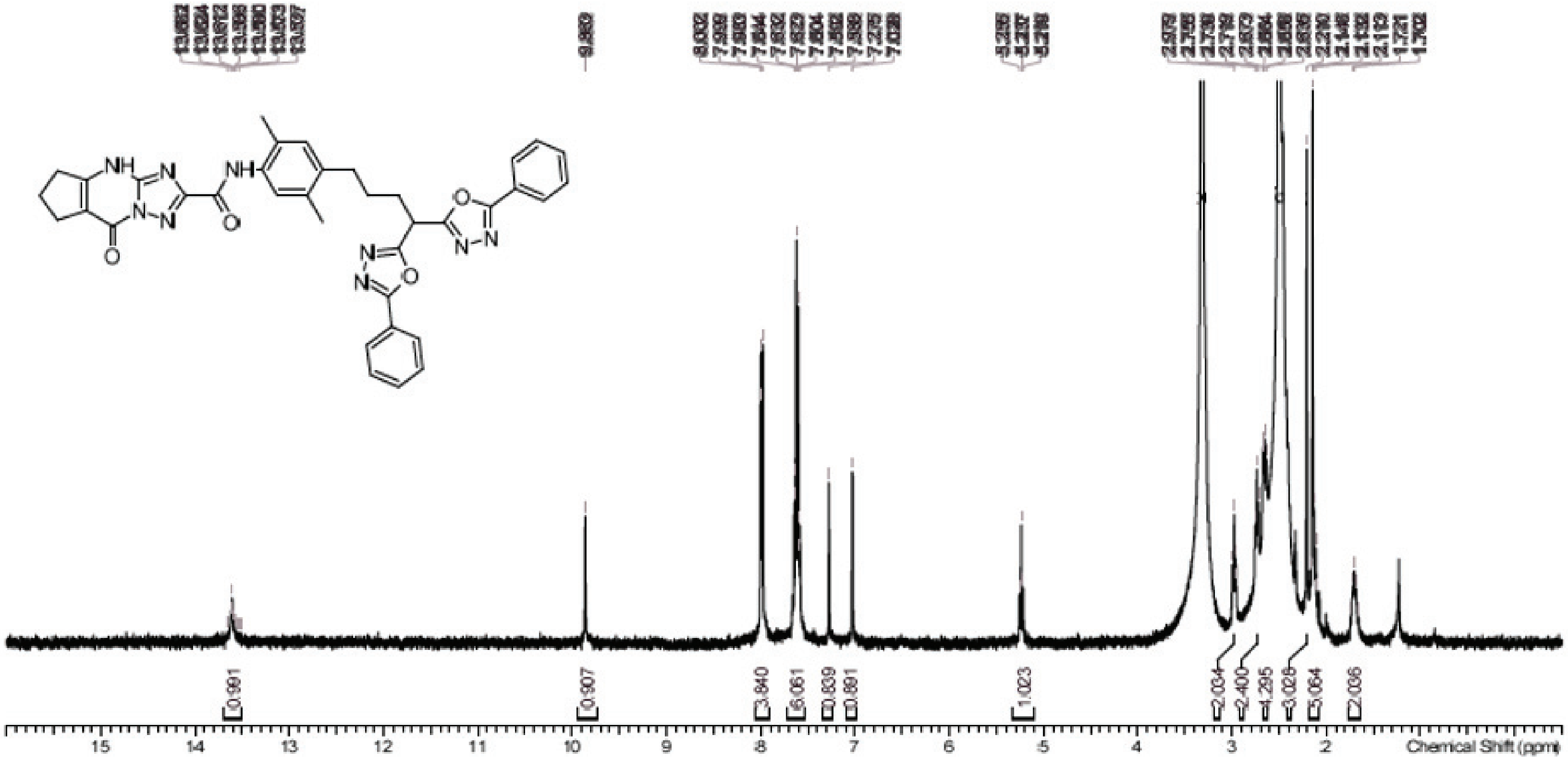

Compound ID: NVS125

EC1995-108-P1A1 DMSO Bruker_CD-C_400MHz

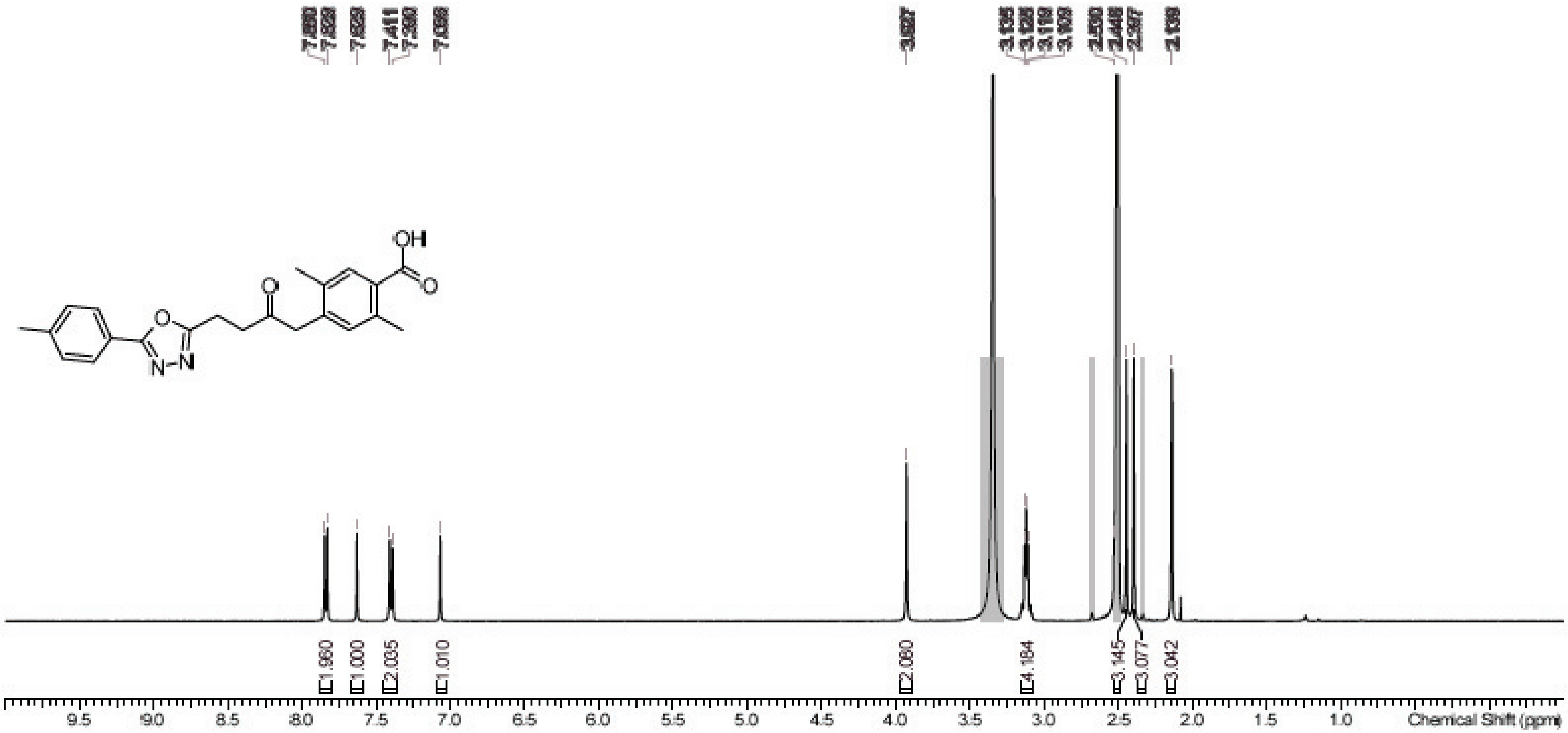

HPLC Chromatograms for NGI03, URD001 & NVS125

HPLC Report

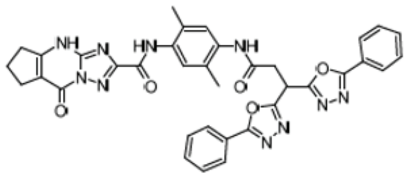

Chromatogram

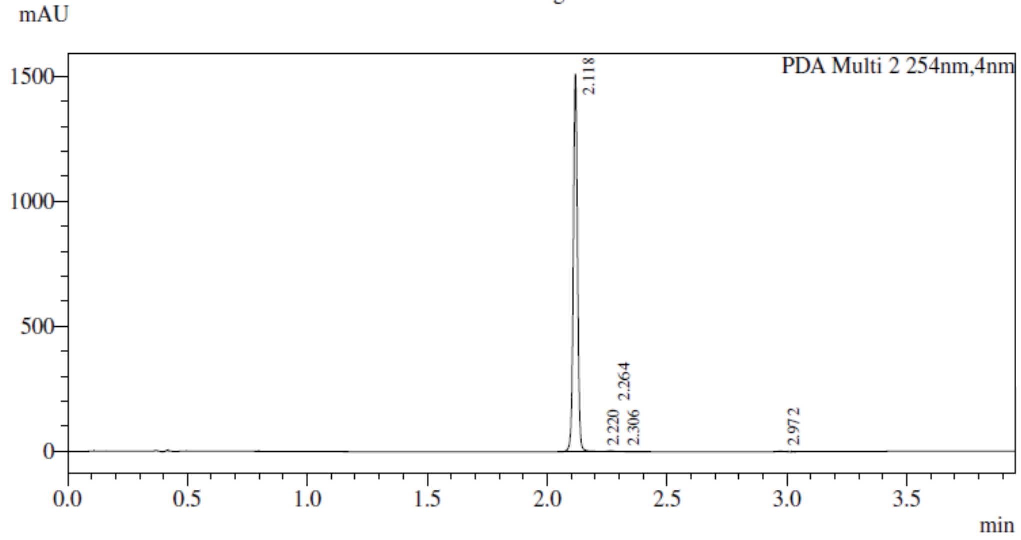

Compound ID : NG103
Sample ID : EC3153-42-P4B1
Injection Vol : 5ul
Location : vial89
Acq Method : D:\Method\10-80AB_4min.lcm
Org DataFile : D:\DATA\2021\2110\211019\EC3153-42-P4B1.lcd
Injection Date : 2021/10/19 11:02:54
Instrument : CAS-CD-HPLC-C

Integration Result

PDA Ch2 254nm

Peak#	Ret. Time	USP Width	Height	Height%	Area	Area%
1	2.118	0.031	1509262	99.427	1839819	99.213
2	2.220	0.417	1486	0.098	1565	0.084
3	2.264	0.041	4374	0.288	7950	0.429
4	2.306	0.093	1406	0.093	3233	0.174
5	2.972	0.035	1425	0.094	1843	0.099
Total			1517953	100.000	1854410	100.000

HPLC Report

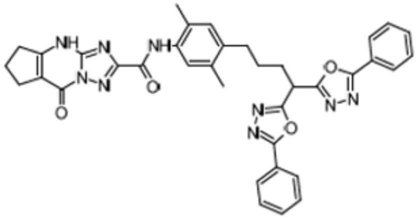

Compound ID : URD001
Sample ID : EC6016-36-P1B3
Injection Vol : 1ul
Location : vial24
Acq Method : D:\Method\10-80AB_4min.lcm
Org DataFile : D:\DATA\2022\2201\220125\EC6016-36-P1B3.lcd
Injection Date : 2022/1/25 14:14:57
Instrument : CAS-CD-HPLC-C

Chromatogram

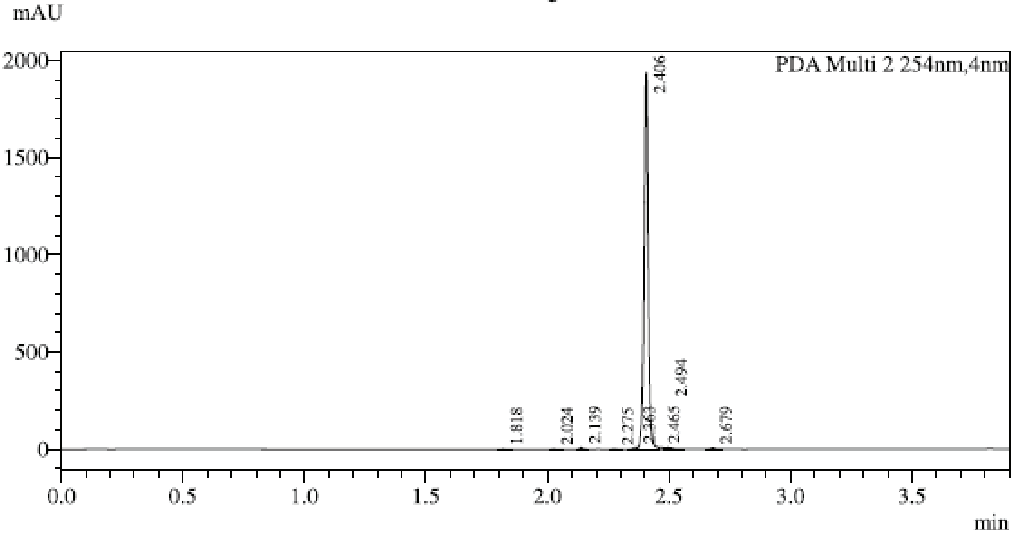

Integration Result

PDA Ch2 254nm

Peak#	Ret. Time	USP Width	Height	Height%	Area	Area%
1	1.818	0.027	1179	0.060	1246	0.054
2	2.024	0.027	1241	0.063	1395	0.060
3	2.139	0.027	8340	0.422	8497	0.367
4	2.275	0.027	1014	0.051	1074	0.046
5	2.363	0.070	8558	0.433	9434	0.408
6	2.406	0.029	1932924	97.799	2256333	97.504
7	2.465	0.086	9911	0.501	12889	0.557
8	2.494	0.113	7632	0.386	16104	0.696
9	2.679	0.032	5636	0.285	7122	0.308
Total			1976433	100.000	2314094	100.000

HPLC Report

==

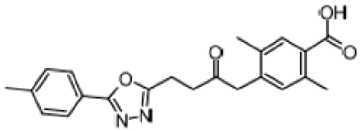

Chromatogram

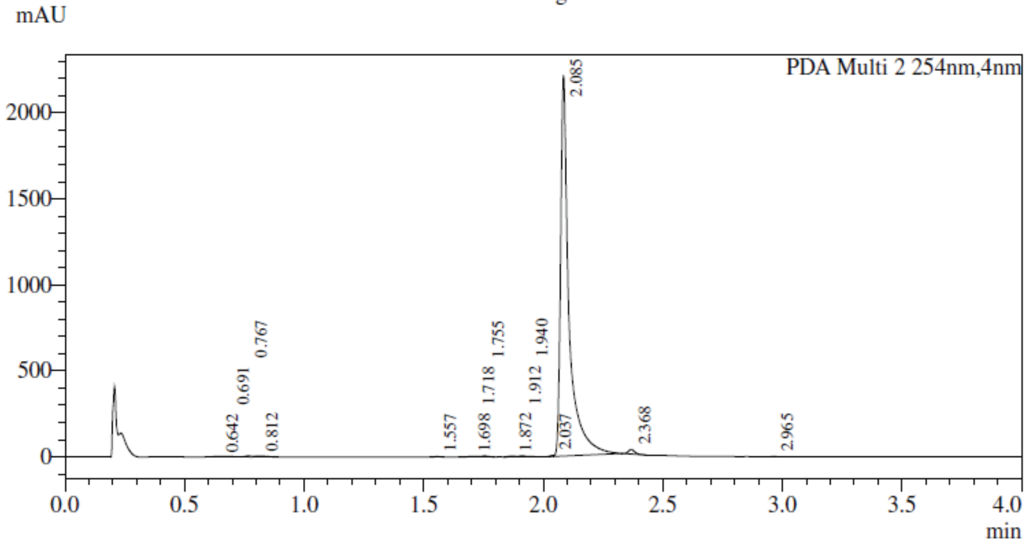

Compound ID : NVS125
Sample ID : EC1995-108-P1C2
Injection Vol : 2ul
Location : vial30
Acq Method : D:\Method\10-80AB_4min.lcm
Org DataFile : D:\DATA\2021\2108\210818\EC1995-108-P1C2.lcd
Injection Date : 2021/8/18 14:44:22
Instrument : CAS-CD-HPLC-C

==

Integration Result

==

PDA Ch2 254nm

Peak#	Ret. Time	USP Width	Height	Height%	Area	Area%
1	0.642	0.251	1403	0.061	4039	0.072
2	0.691	0.657	1627	0.071	4913	0.088
3	0.767	0.058	5985	0.261	10992	0.196
4	0.812	0.092	6016	0.263	19451	0.347
5	1.557	0.040	2706	0.118	4177	0.075
6	1.698	0.023	3133	0.137	2812	0.050
7	1.718	0.071	4965	0.217	9027	0.161
8	1.755	0.049	6648	0.290	11477	0.205
9	1.872	0.062	4993	0.218	10831	0.193
10	1.912	0.047	7002	0.306	11932	0.213
11	1.940	0.353	2696	0.118	4032	0.072
12	2.037	0.071	5419	0.236	5920	0.106
13	2.085	0.051	2210828	96.480	5451163	97.290
14	2.368	0.048	26756	1.168	49891	0.890
15	2.965	0.045	1311	0.057	2349	0.042
